# Common clinical blood and urine biomarkers for ischemic stroke: an Estonian Electronic Health Records database study

**DOI:** 10.1186/s40001-023-01087-6

**Published:** 2023-03-25

**Authors:** Siim Kurvits, Ainika Harro, Anu Reigo, Anne Ott, Sven Laur, Dage Särg, Ardi Tampuu, the Estonian Biobank Research Team, Kaur Alasoo, Jaak Vilo, Lili Milani, Toomas Haller, the PRECISE4Q consortium

**Affiliations:** 1 Estonian Genome Center, Institute of Genomics, University of Tartu, Tartu, Estonia; 2 Institute of Computer Science, University of Tartu, Tartu, Estonia; 3 Software Technology and Applications Competence Center, Tartu, Estonia

**Keywords:** Ischemic stroke, Electronic health records, Population health, Machine learning

## Abstract

**Background:**

Ischemic stroke (IS) is a major health risk without generally usable effective measures of primary prevention. Early warning signals that are easy to detect and widely available can save lives. Estonia has one nation-wide Electronic Health Record (EHR) database for the storage of medical information of patients from hospitals and primary care providers.

**Methods:**

We extracted structured and unstructured data from the EHRs of participants of the Estonian Biobank (EstBB) and evaluated different formats of input data to understand how this continuously growing dataset should be prepared for best prediction. The utility of the EHR database for finding blood- and urine-based biomarkers for IS was demonstrated by applying different analytical and machine learning (ML) methods.

**Results:**

Several early trends in common clinical laboratory parameter changes (set of red blood indices, lymphocyte/neutrophil ratio, etc.) were established for IS prediction. The developed ML models predicted the future occurrence of IS with very high accuracy and Random Forests was proved as the most applicable method to EHR data.

**Conclusions:**

We conclude that the EHR database and the risk factors uncovered are valuable resources in screening the population for risk of IS as well as constructing disease risk scores and refining prediction models for IS by ML.

**Supplementary Information:**

The online version contains supplementary material available at 10.1186/s40001-023-01087-6.

## Introduction

Stroke is playing a major role in affecting not only the people’s health but also the economy. With a worldwide incidence of 12.2 million, and 6.55 million deaths in 2019, it is the second leading cause of death [1]. Stroke survivors often face long hospitalization and rehabilitation programs and many are unable to return to normal lifestyles. Ischemic stroke (IS, abbreviations in Additional file 1: Table S1) is the main form of stroke (62.4% of all cases) resulting from cerebral ischemia (insufficient blood flow, frequently with blockage of blood vessels) [1]. IS can be classified into 5 subtypes: large-artery atherosclerosis, cardioembolism, small-vessel occlusion, stroke of other determined etiology, and stroke of undetermined etiology [2].

The leading known risk factors for stroke are high systolic blood pressure and body mass index, high fasting plasma glucose concentration, and ambient particulate matter pollution [1]. The heritability of stroke is estimated to be 30–40% [3], and a list of IS-associated genomic markers has been identified [4–6]. Several blood parameters are associated with stroke, such as creatinine, lymphocyte, and monocyte counts [7, 8]; hematocrit and hemoglobin [9]; platelet count and mean volume [10], and eGFR [11]. More recently, proteinuria [12] and urine pH [13, 14] have been shown to play a role in stroke.

Many biomarkers are routinely measured from blood or urine for a large number of individuals and could be used in prevention efforts with only a relatively small additional cost. Although the population-level markers may not be optimal for predicting a disease for a specific individual, their abundance makes them valuable for population-level screening programs, as even small effects add up to significant differences. Stroke incidence is rising and early warning markers that are easy to detect and readily available can save lives [15].

The clinical parameters (CPs) from nation-wide healthcare facilities were not retrievable for research until the advent of digitizing the medical system. Estonia’s Electronic Health Records (EHR) database stores medical information for the procedures, carried out in the hospitals and primary care facilities, and the corresponding epicrises data [16, 17]. As all Estonian hospitals use the EHR system this database has population-wide coverage.

The Estonian Biobank (EstBB) encompasses genetic and medical data for 20% of Estonia’s adult population and represents well the whole Estonian population [16]. The work of EstBB is governed by The Human Gene Research Act (HGRA) [18]. All participants of EstBB have consented to using their data anonymously for research purposes and to enrich their health records using national health registries and databases. This task is performed regularly, including updates from the central EHR database [19]. As a result, the available number of data layers from EHR increases significantly when combined with the rich dataset of EstBB which includes traits, such as medications used, ICD-10 codes, diet, physical activity, self-reported health issues, different molecular phenotypes, and many more.

We and others have developed methods not only for extracting medical data from numerical fields of EHRs but also to mine them from free text fields while following all ethical guidelines [20, 21]. Not only can it be used in conjunction with other data layers but also large-scale longitudinal studies can be planned to reveal trends.

**Fig. 1:**
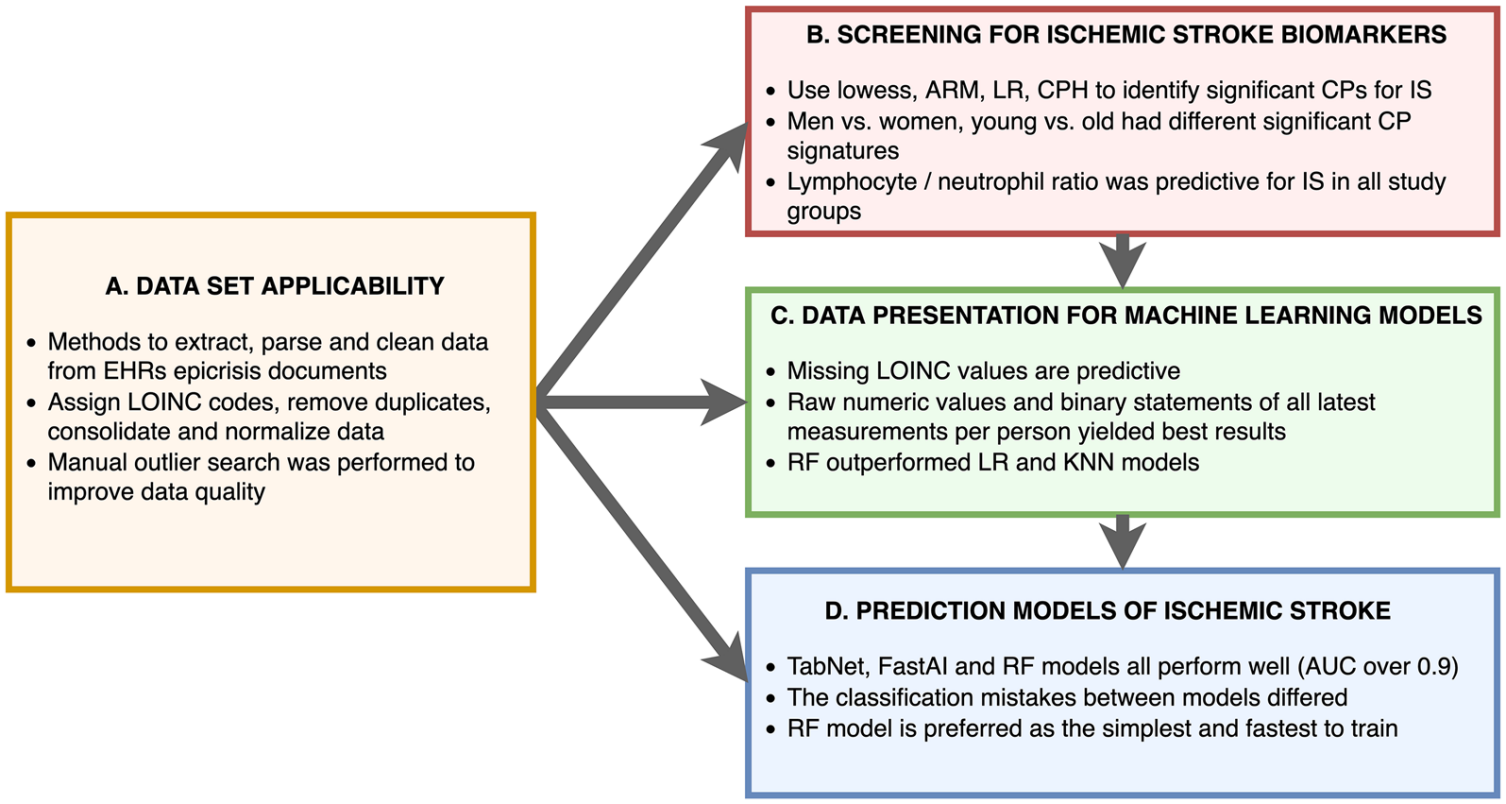
Workflow of the article highlighting the main deliverables. The steps A to D correspond to paragraphs in the Methods and Results sections

Here, we present our research on CPs from blood and urine, as recorded in the EHRs, together with additional medical data from EstBB with the intent to evaluate them as early warnings for IS (Additional file 1: Table S2). We are targeting the following issues:

- applicability of a general country-wide medical database (EHR) for studying IS: determining the optimal ways to curate and present the data for analysis, establishing analytical pipelines;
- screening the EHR dataset for new medical markers for IS or validating known markers as predictors;
- comparing different ways to prepare data for analysis and evaluating this with ML algorithms: logistic regression (LR), *K*-nearest neighbors (KNN), and random forests (RF);
- testing whether modern deep neural network (DNN) methods (TabNet, FastAI tabular) outperform a benchmark RF in predicting IS [22, 23] (Fig. 1).

Our results confirm several previous findings and suggest that EHRs together with the proposed methodologies have a clear potential for assessing the risk of IS. We expect this to advance population-wide prediction of IS in Estonia and elsewhere (Additional file 1: Supplementary figures, tables and text).

## Methods

### Part A: EHR data extraction and cleaning

We define ischemic stroke as ICD-10 code I63 (cerebral infarction) diagnosed by medical specialists and available through EHR or EstBB databases. The clinical chemistry and other laboratory data used originate from EHRs—a data repository coordinated by the Estonian Health and Welfare Information Systems Centre [24]. These records are retrieved from multiple independent laboratory information systems, but a universal LOINC coding system is used [25]. The EHR database was compiled in batches. The current batch (spanning February 2004–March 2020) was prepared specifically for IS (both cases and controls) and it contained data from tabular fields and free text fields for 2250 case and 8296 control candidates with 1540 different LOINC codes (Additional file 1: Text S3). It contained 2.02 million clinical blood or urine measurement entries with attributable LOINC codes [each LOINC represented on average 1309 times (median 12)], ranging from 1 to 64,160; each person had on average 191 LOINC codes (median 96), ranging from 1 to 6166. Several additional steps ensured the quality of the final dataset before subjecting it to downstream analysis (Additional file 1: Text S4).

Four healthy controls were found for each case (by a custom C++ script) to meet the following criteria: (a) matching sex, (b) same or closest possible birth year, (c) no I63 diagnosis in any available database, and (d) no mention of the following ICD-10 diagnoses–– I60 (subarachnoid hemorrhage), I61 (intracerebral hemorrhage), I62 (other non-traumatic intracranial hemorrhage), I64 (stroke, not specified as hemorrhage or infarction)––and/or the following medications (ATC codes)–– B01 (antithrombotic agents), M01A (anti-inflammatory and antirheumatic products, non-steroids), M01BA03 (acetylsalicylic acid and corticosteroids), N02BA (salicylic acid and derivatives).

In case of multiple incidences only the first IS episode was considered and only the CP measurements before the first IS were incorporated (for controls this was the time of the first IS of their matched cases) unless otherwise specified. After all quality control steps 950 I63 cases, 3800 controls, and 145 high quality CPs (defined via LOINC codes) remained. Some CPs had multiple units—these were treated as separate CPs for downstream analyses as no unit conversions were performed to reduce batch effects and technical uncertainties. The less common units typically accounted for 0.1–2.7% of all entries and they never turned out significant in any analyses.

Five different data subsets were constructed from among the total group of matched cases and controls by transferring each case (1): control (4) quintuplex to new sub-groups (*n*_total in parentheses)––(a) all individuals (*n* = 4750), (b) men (*n* = 1925), (c) women (*n* = 2824), (d) young (≤ 60 years old, *n* = 850), (e) old (> 60 years old, *n* = 3900). The CP values were adjusted for sex and age using the *z*-scores unless otherwise specified. For experiments utilizing the 1000-day window before IS the cases and controls were used in a 1:3 ratio because of the limitations imposed by the smaller number of young subjects. For the ML models the cases and controls needed to have at least 10 different LOINC code CP measurements available, with the measurement date within 1000 days before the occurrence of IS, thus resulting in 749 cases and 2033 controls. The cleaning process produced a tabular dataset used for ML models such that every column was a CP and its value the most recent measurement.

The EstBB database has been described elsewhere [16, 19]. Briefly, it is a general population-based biobank (University of Tartu) containing many layers of data for 200,000 inhabitants of Estonia (20% of the adult population) over 18 years of age. A comprehensive questionnaire (objective information, physical activity, diet, etc.) is filled in together with a primary agreement and renewed when joining additional research projects. EstBB also stores DNA, plasma, and white blood cell samples for the participants. The database is updated by regular linking to the national health databases (Additional file 1: Fig. S5). This has enabled us to complement the primary data layers with a list of additional datasets, including molecular parameters.

### Part B: Screening for IS markers

Association Rule Mining (ARM) was performed with an a priori approach making use of FP Growth Algorithm (custom implementation in C++) [26]. Only the latest data point was used for each CP for each individual. The values were adjusted for sex and age and represented as the residuals of the linear model in *z*-scores. Only the high values (*z*-score > 1) and low values (*z*-score < − 1) were used to code all items as “low” or “high.” ARM was performed for each cases group of size *N*. Then ARM was performed 20 times for each controls group of size *N* compiled randomly from selection size *M*, where *M *≈ 5 × *N*. Association rules (ARs) that had a higher support for cases than any of the 20 controls iteration were selected for further filtering and testing (Additional file 1: Text S6).

For locally weighted scatterplot smoothing (lowess) analysis the values were adjusted for sex (unless sexes analyzed separately) and age and represented as the residuals of the linear model in *z*-scores. These *z*-score values were used by R function lowess (default values) to generate the curves. The curves were evaluated visually for the following parameters: trend start time (defined as the time from which the cases’ *z*-score value had continuously the opposite sign as compared to that of the controls’), trend direction (positive or negative with respect to approaching IS diagnosis or observation stop time), overall assignment confidence (as a binary value: lower 50% or higher 50%). Trend was considered significant if the *z*-score change over the observed length of the trend was larger than 0.1 units and the lowess curve was monotonous throughout the trend length.

For logistic regression (LR) the values were transformed to achieve normal distribution and remove outliers (Additional file 1: Text S7). LR was carried out with R glm function glm(IS ~ value + sex + age). Adjustment for sex was not done when sexes were analyzed separately.

Cox Proportional Hazards (CPH) analysis was carried out with R. Only the last data point was used for each CP for each individual. Sex and age adjusted values were divided into three intervals based on *z*-scores: (− ∞, − 1), [− 1, 1], (1, ∞). Kaplan–Meier (K–M) graphs were created with R (Additional file 1: Text S8).

When assessing the CP ratios the individual parameters (to be used in ratios) were automatically selected so that their dates were as close to one another as possible.

### Part C: Comparing different data representation types

The LR, KNN, and RF were used to evaluate different input data preparation methods. The prediction models were compared to baseline models involving only sex and age as the inputs. All methods were performed with Python 3.7 using Pandas [27], NumPy [28], and scikit-learn [29] libraries. The search for optimal hyperparameters was done on a separate dataset that was not used in training or testing of ML methods. In total 79,000 CP measurements were available for cases and 144,000 for controls. The separate dataset for search of hyperparameters represented 5% of the total CP measurements, leaving 95% of the cleaned dataset (final dataset) for training and testing sets. The final dataset was divided into training set of 95% and test set of 5%. For cases, only the CPs within a 1000-day window prior to the first IS were used for input.

### Part D: Constructing prediction models

TabNet is a state-of-the-art DNN for tabular data modeling which uses sequential attention to choose features at each decision step and enables both local and global interpretability [23]. FastAI tabular learner is the default neural network architecture proposed by the FastAI library for analyzing tabular data. Both of the DNNs were implemented using Python’s FastAI library (version 2.3.1) [22] (Additional file 1: Text S9). RF was implemented using Python (v. 3.7.10) sklearn library (v. 0.22.1) [30].

For the comparison of RF and DNN performance, the last observation carried forward approach was used: the latest available LOINC and ICD-10 values were used for every person. Similarly to the previous analysis only analytes within 1000 days prior to the first IS were used for input. The ICD-10 predictive feature could be any ICD-10 code except for I60, I61, I62, I63, and I64. These codes were removed from the ICD-10 predictive feature.

In addition to measuring the predictive ability, also the feature importance for all 3 models were found with methods appropriate for each model type. For the RF model and TabNet the built-in feature importance methods were utilized. The Gini importance for the RF and sparse features selection-based method for TabNet were used. Because the FastAI tabular has no built-in feature importance methods, a permutation importance method was implemented.

All computations and file manipulations if not otherwise specified were carried out with R (v. 4.0.3), Python (v. 3.7.10) or C++/Qt (v. 4.3 or higher). All *p*-values were calculated through two-tailed testing.

Personal-level data were available for research only in the pseudonymized form to protect the privacy of the participants. Best practices were used throughout the project to ensure no ethical compromises. This study has been approved by the Research Ethics Committee of the University of Tartu, and Estonian Committee on Bioethics and Human Research.

## Results

### Part A: EHR data collection, preprocessing, cleaning

Our first goal was to establish a sequence of steps to retrieve pseudonymized EHR data for research. This included obtaining the necessary ethics committee permits and ensuring that all work followed the HGRA [18]. Data retrieval was an elaborate process consisting of multiple stages (Additional file 1: Text S3). We initially aimed to demonstrate the usability and quality of the EHR dataset for studying IS, a representative of a common disease, to pave the road for using these data in future projects as well. As the EHRs are mined from sources of various structure and quality, we needed to establish a semi-automatic pipeline for its quality control (QC) and formatting (Additional file 1: Text S4, Fig. S1). We performed cross-checking between EHR and EstBB databases and retained only the individuals whose IS status was the same in both. Additionally, the EstBB provided lifestyle information and data about other comorbidities for the merged final dataset.

We started with 950 IS cases (40.5% men) and matched 4 controls to each from among 7398 healthy individuals, achieving a 100% perfect fit between cases and controls for sex and a 98.8% fit for age. The mean age of cases (71.32 ± 12.79 SD) was very similar to that of controls (71.34 ± 12.74 SD). An age split at 60/61 was used to separate young individuals from old due to the small number of available young IS cases (Table 1).

**Table 1:**
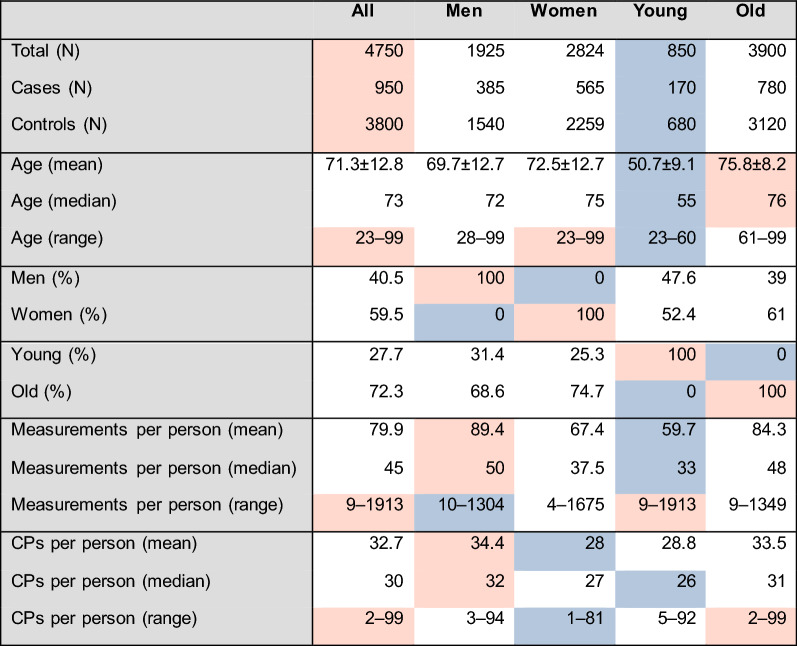
Overview of the 5 sub-groups studied. The lowest value on each row is highlighted in blue, the highest value in red

**Fig. 2:**
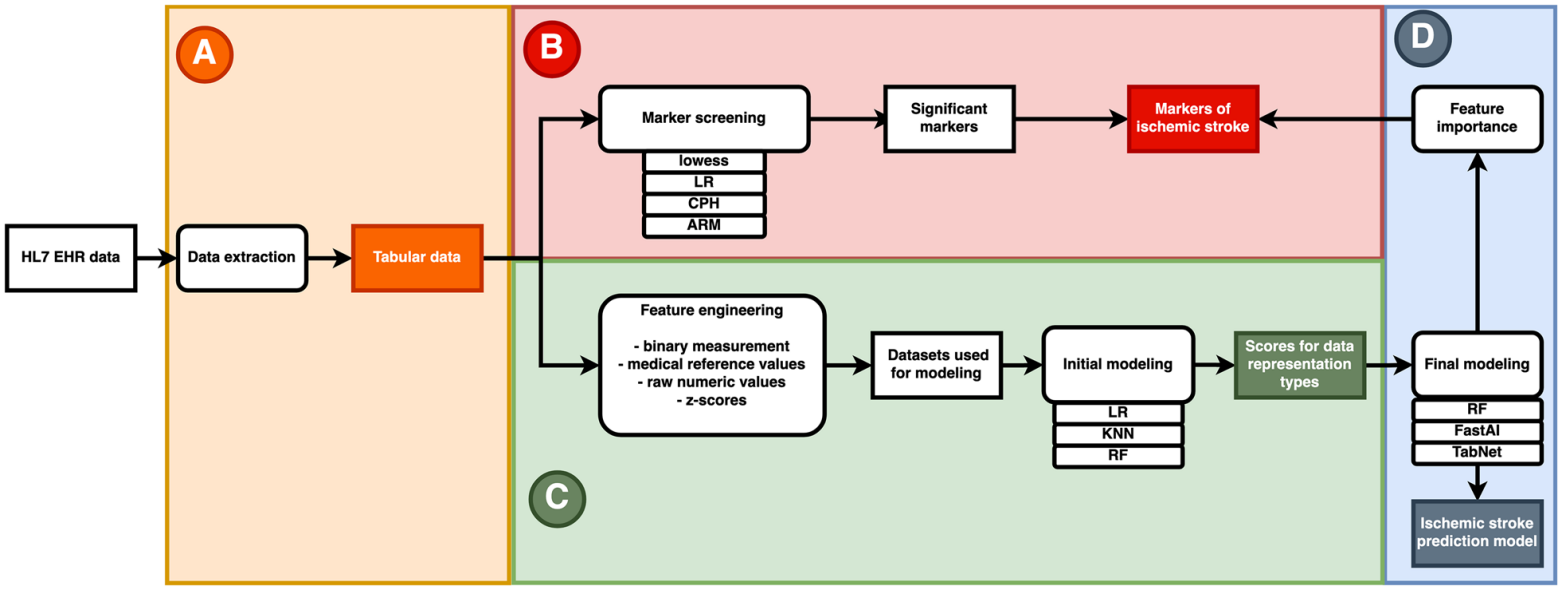
Summary of findings corresponding to the steps A to D explained in Fig. 1

The median number of CPs for each subject varied between 26 and 32, while the age of IS patients varied between 23 and 99 across all sub-groups. The median age difference between the young and old was 21 years (55 vs. 76). The age-specific stroke incidence and mortality rates are known to be higher in men than in women, yet stroke affects a greater number of women because of various comorbidities and sociodemographic factors (e.g., increased longevity) [31, 32]. We also observe that 31.4% of males in our study were classified as young cases of IS compared with 25.3% of females. The average age of male IS patients was 69.7 (median = 72) as opposed to 72.5 (median = 75) for women (Table 1, Fig. 2).

### Part B: Screening markers for IS

In the first set of experiments we used standard methods to establish associations between IS and CPs: ARM, lowess, LR, CPH (Fig. 1). ARM was performed for the 1000-day window before the incidence of IS to detect combinations of CPs that might associate with IS. This yielded 5 relevant ARs after applying stringent filtering criteria (Additional file 1: Text S6), all consisting of two items. Of these rules only one was not obvious and could be detected in all sub-groups: B.Lymph.%_LOW + B.Neut.%_HIGH (full names of CPs in Additional file 1: Table S2). We further tested this rule to show that the support ratio of observed/predicted was always significantly over 1 and ranged between 2.2 (young) and 5.97 (women). Thus, for all groups the association between the two individual items was at least two times higher than expected if these items were associated by chance (Additional file 1: Table S10).

We studied the CPs by combining all individual measurements (adjusted for sex and/or age and as *z*-scores) for each CP and constructing lowess curves (Additional file 1: Fig. S11). The trends in lowess curves before the onset of IS can be used to pinpoint predictive changes in CPs. Several CPs showed trends 500–3500 (most commonly 2000) days before the onset of IS. Overall hemoglobin, cholesterol, and blood clotting parameters stood out. The highest confidence positive trends with respect to IS for all sub-groups were B.RDW.CV, B.MPV, B.Neut.#, S.P.Urea, and B.RDW.SD, and negative trends were B.Hct, B.Hb, and B.Lymph.%. The S.P.Crea had a clear positive trend for all but the young. Interestingly all observed cholesterol parameters (S.P.Chol, S.P.LDL.Chol, S.P.HDL.Chol) appeared protective for all sub-groups (only S.P.Chol not so for the young). However, tracking the more informative cholesterol ratios we observed a clear trend (negative) with respect to IS only for the S.P.HDL.Chol/S.P.Chol ratio and only so for men. The ratio of B.Lymph.%/B.Neut.% discovered with ARM correlated negatively with IS. The lowess curves did not show this only for the young. However, some of the potential trends may not have been detected due to the smaller sample size of the young sub-group (Additional file 1: Table S12, Table 2).

**Table 2:**
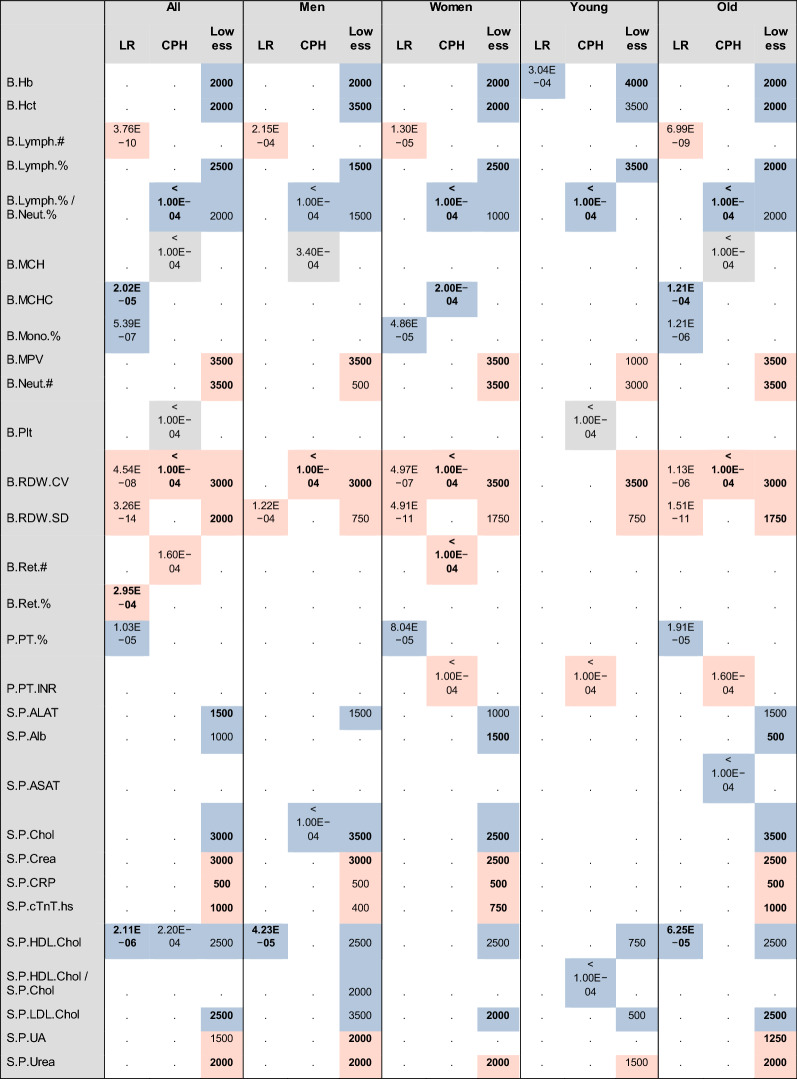
Summary of lowess, LR, and CPH

Table is showing statistically significant *p*-values (LR, CPH) or trend length in days (lowess) in alphabetical order. Pink highlight = positive association with IS, blue highlight = negative association with IS, gray highlight = bimodal association with IS (both low and high concentrations associate with IS better than intermediate values). Bold numbers indicate corresponding absolute effect sizes larger than 0.5 (LR), whether the proportionality of hazards was confirmed (CPH) or higher than average certainty of assigning the trend (lowess)

We ranked the CPs for a 1000-day time frame before the initial episode of IS by LR. After Bonferroni correction for multiple testing 9 parameters were significant in at least one sub-group: B.RDW.SD, B.Lymph.#, B.RDW.CV, B.Mono.%, S.P.HDL.Chol, P.PT.%, B.MCHC, B.Ret.%, B.Hb. The total cohort shared most similarity with the old sub-group (probably due to the predominantly advanced age of typical cases) with only B.Ret.% barely not showing significance in the old sub-group. The S.P.HDL.Chol appeared protective against IS and met the significance threshold for men but not for women. Its effect size was among the highest out of all significant hits. We detected 3 more CPs as significant for women but not for men: B.RDW.CV, B.Mono.%, P.PT.%. The young sub-group showed an entirely different signature with the B.Hb as the only significant hit. Again, this highlights the underlying differences between the young age and advanced age IS [33]. We performed LR on three CP ratios (S.P.HDL.Chol/S.P.Chol, S.P.LDL.Chol/S.P.Chol, B.Lymph.%/B.Neut.%). None of them passed the Bonferroni threshold (Additional file 1: Table S13, Table 2).

CPH was performed using the most recent measurement for each parameter for each individual. Three intervals were created based on *z*-score: (− ∞, − 1), [− 1, 1], (1, ∞). The Kaplan–Meier (K–M) curves indicate major differences between the tested sub-groups. It is noted that Hazard Ratio (HR) is meaningful only if the proportional hazards assumption is met, i.e., the curve for the middle group according to the *z*-score also appears in the middle in the K–M graphs (Additional file 1: Fig. S14). These parameters showed significant *p*-values (Bonferroni-corrected threshold 3.4 × 10^–4^ at the significance level of 0.5) together with proportionality of hazards: B.RDW.CV (for all, men and old), B.Ret.# [for women and old (*p*-value 5.4 × 10^–4^)], B.MCHC (for women) (Additional file 1: Table S15). The first two were associated with an increased risk for IS (HR of B.Ret.# for women was as high as 5.6), while B.MCHC had the opposite trend. The S.P.Chol exhibited a protective HR of 0.75, but the hazards were proportional only for old and the *p*-value was just outside of the Bonferroni threshold. The *p*-values for B.Plt and B.MCH were very low but the hazards were never proportional. The corresponding K–M graphs suggest bimodal behavior for these CPs as both the higher and lower concentrations associated with the increased risk for IS (Additional file 1: Figs. S16 and S17). Only B.Hb qualified as having proportional hazards for the young sub-group. Its *p*-value of 2.2 × 10^–3^ was, however, outside of the Bonferroni threshold. It is still noteworthy that B.Hb was also the only significant hit for this group based on LR.

We analyzed the same 3 CP ratios by CPH as was done by LR (Additional file 1: Table S15). The S.P.HDL.Chol/S.P.Chol showed significant *p*-value for the young; however, the K–M graph did not confirm the proportionality of hazards (Additional file 1: Fig. S18). This ratio had a nominal significance for the all and men sub-groups, but notably not so for women. Interestingly, the B.Lymph.%/B.Neut.% showed the highest detectable level of significance for all groups. The corresponding K–M graphs all confirmed proportionality of hazards (borderline for the men sub-group) with the HR values ranging from 0.59 to 0.81 (Additional file 1: Fig. S19).

Summary of the tests carried out (Fig. 2) outlines the most significant CPs for all cohort sub-groups. All analytical methods always showed the same effect direction for all CPs (Table 2).

### Part C: Comparing different data representation types

In the second set of experiments we tested several input representation methods to examine the predictive value of EHR data for IS (Fig. 1). We wished to determine: (1) if the fact of the measurement itself was informative regardless of the numerical value, (2) whether the use of existing clinical reference values could improve predictions, (3) the effect of adjusting the values for sex and age, and (4) whether the data should be treated as measurement based or patient based. LR was selected as the benchmark method to test the applicability of linear models to these data. KNN with Euclidean distance was the second model where the value for *K* was chosen by multiple pretests. RF was selected because it has been effective on various medical datasets, especially in handling noise [34]. The number of trees was found by examining various options on a separate dataset that was not used for training or testing. The rest of the hyperparameters for KNN and RF were not tuned further and the default values of the scikit-learn version were used.

The EHR dataset can be transformed into the tabular form in two ways: measurement based or individual based. For the first, all CP measurements were treated as independent data points, allowing multiple data instances for each person. For the individual-based structure only the latest CP value was used for each individual. Thus, each individual was represented in the dataset only once.

Five different approaches were tested for the LR, KNN, and RF comparison:

- using binary statement of measurements (the ML input consisted only of the statements of 1 and 0; whether CP was measured or not),
- using binary statements balanced by selecting entries with comparable number of statements in controls and cases to limit the CP bias,
- using the medical reference values: the CP values categorization (categorical feature) based on whether they fell below, within or above the reference range provided by the CP measuring laboratories,
- using the raw values of CP concentrations,
- using the *z*-scores of step (d) raw values adjusted for sex and age.

RF yielded the best results (Table 3). The sex and age only baseline yielded maximum accuracy of 0.72 and precision of 0.66. Medical reference values yielded 0.9 precision and 0.9 accuracy. The binary statement of measurements yielded 0.93 precision and accuracy. The AUC scores for binary statement of measurements and medical reference values approaches were 0.93. No approach resulted in AUC below 0.9. This suggests that the fact of having certain analytes measured by medical personnel contains enough information for a good prediction. However, since the medical reference values approach yielded similar results, it is possible that ML methods perform better if optimized further.

**Table 3:**
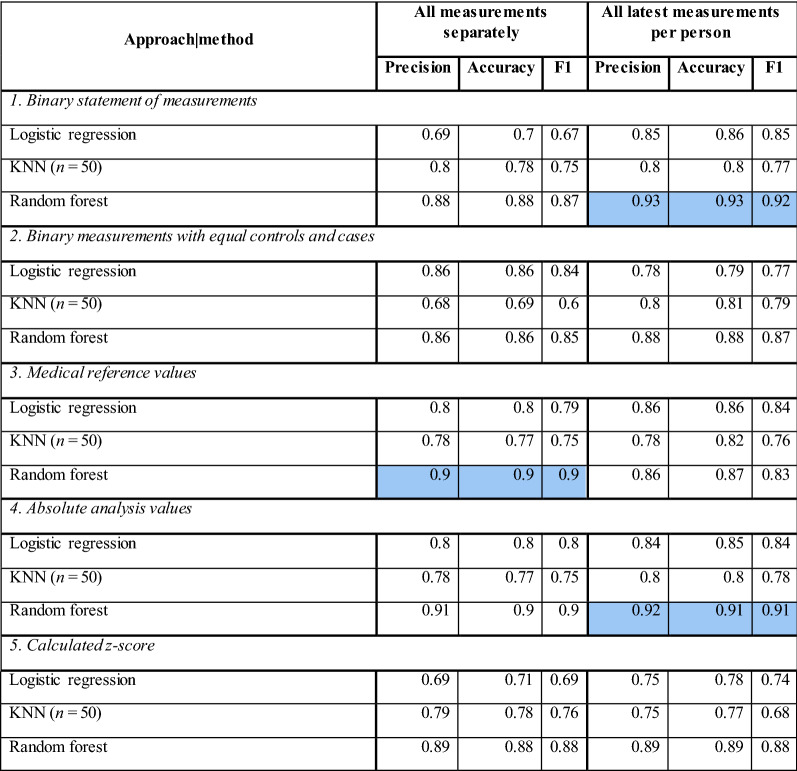
Five different approaches for handling input data

(1) Binary statements whether or not an analyte was measured. (2) Binary statements were equalized within cases and controls. (3) Medical reference values were utilized; each value was marked as below, within, or above the reference norm. (4) Absolute values were used. (5) *z*-scores were used (adjusted for sex and age)

### Part D: IS prediction models

In recent years, several feed-forward networks have been claimed to outperform tree-based methods when analyzing certain types of tabular data [23]. Here, we selected two promising feed-forward neural networks (TabNet, FastAI tabular learner) and tested whether they outperform the baseline RF model in our IS prediction task (Fig. 1). In this analysis, we proceed with CPs represented by the raw numerical values (Additional file 1: Table S20). This data representation yielded the second best results in Part C but contained more information than the binary observations. Also, the non-stroke ICD-10 codes from previous epicrises were added to the model. As the controls had no I60, I61, I62, I63, and I64 ICD-10 codes, the same codes were also removed from any cases before carrying forward the last observations for features. The codes were one-hot encoded for the RF model, while both DNN methods used an embedding layer for ICD-10 codes. For all three models a grid search algorithm was used to find the best hyperparameters (Additional file 1: Text S9). This hyperparameter search was conducted on the training dataset composed of 90% of the available data using tenfold cross-validation. The hyperparameters yielding the greatest mean AUC value were determined. The final model was then trained on the entirety of the training set (90% of data) using these best hyperparameters and evaluated on the held out set (10% of data) (Additional file 1: Fig. S21).

For RF the mean AUC score for the best set of hyperparameters on the tenfold cross-validation was 0.92 (SD 0.02), which translated to AUC = 0.94 of the final model. The FastAI tabular model achieved AUC of 0.86 (SD 0.04) in cross-validation and an AUC of 0.88 on the test data with the final model. TabNet mean AUC score for the tenfold cross-validation was 0.93 (SD = 0.01) and the AUC of the final model was 0.90 on the test dataset (Table 4). Hence, the highest predictive ability on the test dataset was achieved by the RF model (AUC = 0.94). This was surprising given that the DNN approaches were reported as superior for tabular data [23].

**Table 4:**
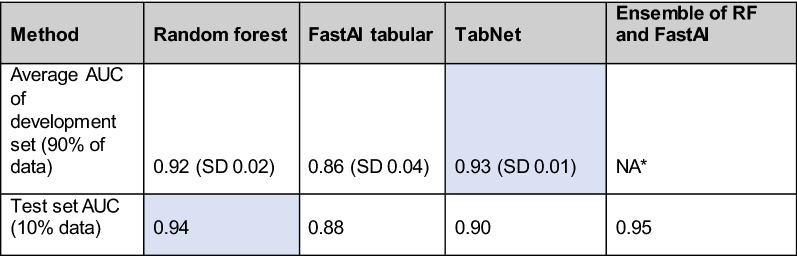
Comparison of the ML methods by AUC

Tenfold cross-validation result with the best hyperparameters found. The ensembling was not performed in the hyperparameter search phase

*The ensembling was not performed in hyperparameter search phase

The error analysis revealed that the DNN and RF models made mistakes on different test dataset instances, revealing that their decision process was different. Ensembling is particularly useful when combining models with uncorrelated mistakes [35]. We manually designed a simple set of ensemble models that, however, offered only minimal improvement (AUC = 0.95) over the stand-alone RF model (AUC = 0.94) (Additional file 1: Table S22).

**Fig. 3:**
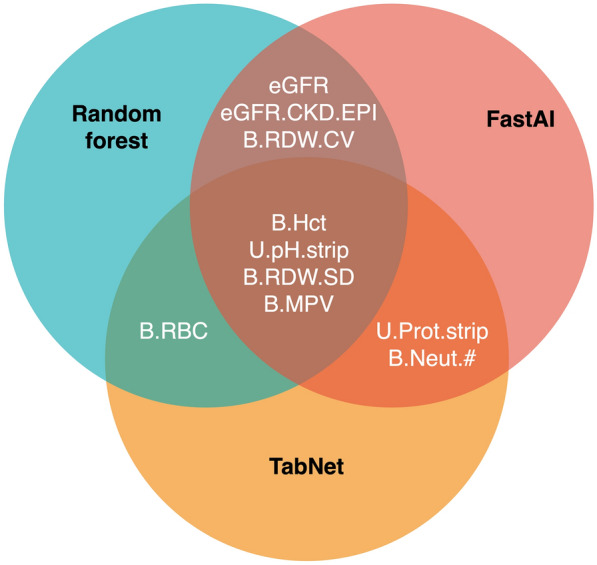
The overlapping CPs found in the top 20 most important features by the 3 models: RF, FastAI tabular, and TabNet

The feature importance analysis of the ML models agreed with the current knowledge of IS risk factors. The most important features according to our models have been previously associated with stroke. Five features (the year of birth, B.Hct, U.pH.strip, B.MPV, and B.RDW.SD) were among the top 20 most important features for all 3 models developed (Fig. 3).

## Discussion

We demonstrated a novel use of an electronic nation-wide healthcare dataset for scientific research through a biobank. Our project served as a successful test for several institutions to work together on medical data while protecting the subjects’ privacy. The rapidly evolving privacy-preserving analysis tools will enable increased secondary use of healthcare data for research purposes [36].

We showed that the EHR database is a valuable and readily usable resource for studying IS risk factors and is projected to have a similar value for studying other diseases (Fig. 2). Our pipeline for preparing and cleaning the EHR data and combining it with additional information from EstBB sets an example for bringing together several large electronic data collections to target one goal.

We started with a wide range of data fields consisting of serum and whole blood samples along with urine and other biofluid analysis results. Treating the CPs as *z*-scores in the association analyses proved very comparable to treating those values relative to medically set ranges.

Lowess analysis, LR, and CPH modeling all uncovered significant hits for IS and confirmed many previous findings while ARM directed our attention to the association of low B.Lymph.% and high B.Neut.% among the IS cases. A meta-view of these results highlights the different risk prediction parameters for different sub-groups (Table 2).

We determined the IS risk factors separately for men and women, as well as for younger and older individuals. Younger and older patients showed a different molecular background for IS [33]. More so than in other groups the young age is associated with low values of hemoglobin and high red blood cells distribution width. Young IS cases are rare and could not be studied separately in depth. The cholesterol parameters exhibited different associations in men and women. We suspected that this effect could be modulated by the usage of statins, aspirin, or other blood thinners. This does not seem to be the case, however, as they show very similar statin usage (52.1% of men, 50.5% of women) according to the EstBB database. Aspirin data are not reliable enough for conclusions because only 1% of men and 1.3% of women show confirmed usage. Individually, the cholesterol parameters had negative association with IS. Typically, the ratio of HDL over total cholesterol is considered informative. Yet this ratio did not confirm the protective nature of HDL cholesterol in the LR or CPH tests.

Association rule mining yielded that a low lymphocyte to neutrophil ratio could be a risk factor for IS. The LR did not confirm this rule after applying Bonferroni correction (the *p*-values were nominally significant for all groups except the old). However, CPH produced significant *p*-values for this rule for all sub-groups and suggested that a high lymphocyte to neutrophil ratio is associated with a lower IS risk (hazard ratios 0.59–0.81). Interestingly the lymphocyte-neutrophil balance has been shown to influence the degree of COVID-19 severity [37], is known as a measure of general inflammation state [38], and has been reported as a prognostic marker for IS [39, 40].

Our results suggest that 5 different health aspects could be compromised for the individual to develop elevated risk for IS: (1) red blood and iron metabolism (B.RDW.CV, B.Hct, B.Hb, B.RDW.SD, B.RBC, S.P.Fer), (2) thrombocytes and coagulation (B.MPV, P.APTT), (3) white blood cells and inflammation (B.Neut.#, B.Lymph.%, B.Segmented.Neut.%, S.P.CRP, B.Lymph.%/B.Neut.%), (4) lipidomics and liver function (S.P.Chol, S.P.LDL.Chol, S.P.HDL.Chol, S.P.ALAT, S.P.ALP, S.P.CA.125, S.P.Alb, B.HbA1c, S.P.HDL.Chol/S.P.Chol), (5) renal function (S.P.Crea, S.P.Urea, S.P.UA, S.P.cTnT.hs, S.P.CA.125, eGFR, S.P.CK).

These 5 above-mentioned pathophysiological conditions before the IS event have been described in literature and can be elaborated further:

1. A rise in the erythrocyte indexes B.RDW.CV and B.RDW.SD and ferritin levels with lowering trends in B.Hb, B.Hct, and B.RBC values before IS diagnosis indicate early hidden anemia. This is characterized by possible anisocytosis (B.RDW.CV, B.RDW.SD) with iron not properly entering the erythrocytes (ferritin) and the hemoglobin oxygen carrier function (B.Hb, B.Hct) operating below normal levels during a prolonged time before the IS onset. The pre-existing anemia was previously shown to cause higher risk for IS and worse IS outcome [9, 41, 42].
2. The positive trend of B.MPV with the onset of IS indicates platelet activation for increased coagulation. Higher B.MPV has been described as a marker of proinflammatory condition of the stroke patients, observable prior to the acute ischemic event [43]. Higher values of B.MPV in the acute phase of stroke have been suggested to indicate early neurological deterioration [44].
3. The lymphocyte–neutrophil imbalance detected before IS reflects chronic inflammation. The immune system has been implicated in the development and progression of common risk factors for stroke [45]. The ratio of B.Lymph.%/B.Neut.% could serve as an early IS biomarker. This has been suggested as useful for assessing acute phase IS and later outcomes [46], sepsis [47], and COVID-19 [48].
4. Dyslipidemia is known as a major risk factor for stroke, including IS [49]. Our dataset provided input for total, HDL, and LDL cholesterol but not enough data for other relevant markers, such as triglycerides, homocysteine, and apoA. We saw weak correlation of all cholesterol types with IS and attribute that to the general frailty due to various comorbidities or advanced age. Of the cholesterol ratios tested only S.P.HDL.Chol/S.P.Chol showed some protective effect and only in men (lowess) or young (CPH) IS patients.
5. High levels of S.P.Crea, S.P.Urea, and S.P.UA together with negative trends of eGFR [50] and S.P.CK indicate impaired renal function. An increase in the levels of S.P.cTnT.hs and S.P.CA.125 also fit in this pattern of moderate renal and hepatic failure.

Further research should go into the causal sequences and relationships between these 5 interrelated conditions preceding IS.

The CP concentration trends were often detectable over 2000 days before IS (Additional file 1: Table S12). This timeframe exceeds the 1-year period typical for finding new clinical markers for IS [51]. Earlier detection options offer advantage because often more serious deviations in CPs reflect changes that have already progressed past where medical attention can help. Since the predictive markers here belong to the commonly administered set of tests, the additional population screening costs are small.

We showed promising predictive value for IS using the ML methods. Sex and age alone showed only weak predictive power. Somewhat surprisingly the binary statement of measurements contained a substantial amount of information for good prediction models (> 0.9 accuracy and precision). This could signify chronic health problems of the future IS patients. As many variables of EHR data were missing it shows that missingness in the EHR variables is not random, but highly associated with patients’ comorbidity data [52]. This suggests that the tests ordered by medical personnel could be sufficient information for an accurate IS prediction model. A relatively small increase in score values was observed when the CP test results were included as input. When comparing LR, KNN, and RF we concluded that RF outperformed other methods and it did so with different data representations. Using raw numeric CP values, the RF resulted in scores above 0.9 proving good applicability of RF on EHR data (Fig. 2).

We also developed and validated ML models to predict IS based on combined EHR and EstBB datasets. The IS could be predicted with excellent performance (AUC was 0.94 for RF and 0.95 for the ensemble model of RF and FastAI). Despite positive results reported in the literature, neither of the DNNs improved the prediction accuracy [23, 53]. We therefore report the simpler and more widely used RF approach as the most promising method for accurate IS classification. The DNN approaches may still prove useful for predicting other diseases—the error analysis showed that DNNs made mistakes in different stages and the importance analysis revealed that DNNs relied more on ICD-10 values. For predicting another disease the decision-making logic of the DNNs might be more suitable and outperform RF.

This work could be improved in several ways. In Estonia, all medical laboratories are obliged to deposit their clinical test results to EHR [54]. Over 20 different laboratories and hospitals perform these tests and they may use different methodologies. Therefore, they must provide the norm and reference values for each test, but they do not always report them to EHR. This may introduce deviations in the standardization steps which translates into poorer input for the prediction models. Secondly, the EHR dataset contains more information for patients who visit doctors more frequently. This can have an effect on our results. Although we use limited pharmaceutical information in our research, this aspect could be improved by incorporating more information about medications used.

We obtained the best predictive results with the most common CPs. With a higher number of rare CPs the results would likely have been different since most ML methods work optimally with a balanced number of parameters. It is possible that with better feature engineering the outcome could improve further.

Finally, because a case–control experimental design was utilized the results are not straightforward to interpret. When developing a clinical risk model one usually considers the target population for the model, time-at-risk, observation time, etc. These parameters would have allowed us to evaluate the applicability of the models better. Regardless, the performance statistics were very encouraging. The focus of this study was to measure the discriminative ability of prediction models (AUC), but the clinical utility of the models still needs to be assessed before the models are ready for medical use. Further research toward fine-tuning more advanced prospective models for clinical use can include our findings as input, while each of these models requires additional evaluations.

## Conclusions

This project has been an introduction to more in-depth analysis of predicting IS and also other common diseases in the Estonian population. Our study serves as an example of how to screen an already existing EHR datasets for CPs to be incorporated into general risk calculations for IS in the future. We established several trends in common clinical parameter changes that can be used as early warning signals for IS. Our ML models were able to accurately predict future IS. It is of paramount importance to compile the current understanding and generate new knowledge to proceed to generating multi-level risk models for predicting IS and other common diseases at the population level. Therefore, steady work is still required for applying the scientific results to benefit public health.

## Supplementary Information

**Additional file 1:** **Table S1.** Abbreviations used in the article. **Table S2.** Clinical parameters mentioned in the article. **Text S3.** Raw data extraction workflow. **Text S4.** Semi-automatic pipeline for cleaning raw tabular EHR data for downstream analyses of IS. **Fig. S5.** Sources for phenotype information from the EstBB. **Text S6.** Filters used in Association Rule Mining (ARM) to identify potentially interesting rules for further testing. **Text S7.** R script to transform numerical clinical data for Logistic Regression (LR). **Text S8.** R script used for Cox Proportinal Hazards (CPH) model and Kaplan-Meier (K-M) graphs. **Text S9.** Deep neural networks (DNN) implementation. **Table S10.** Five association rules (ARMs) identified. **Fig. S11.** Lowess curve examples. **Table S12.** Summary of lowess. **Table S13.** Summary of Logistic Regression (LR). **Fig. S14.** Kaplan-Meier graphs for CPs with P<0.001 and proportional hazards. **Table S15.** Summary of Cox Proportional Hazards (CPH) model. **Fig. S16.** Kaplan-Meier graphs for B.Plt. **Fig. S17.** Kaplan-Meier graphs for B.MCH. **Fig. S18.** Kaplan-Meier graphs for S.P.HDL.Chol/S.P.Chol. **Fig. S19.** Kaplan-Meier graphs for B.Lymph.%/B.Neut.%. **Table S20**. Data sources for ML models. **Fig. S21.** Workflow of ML model creation and testing. **Table S22.** The ML ensemble models tested.

## Data Availability

The datasets generated and analyzed during the current study are not publicly available due to the limitations set by the ethics regulations and the legal framework, but the data can be available in the pseudonymized form through the Estonian Biobank data release system when complying with all data release and ethics regulations and the Human Genes Research Act which regulates data release from the Estonian Biobank.

## Abbreviations

AR: Association rule
ARM: Association rule mining
ATC: Anatomical therapeutic chemical (code)
AUC: Area under the curve (here same as AUROC)
CP(s): Clinical parameter(s)
CPH: Cox proportional hazards model
DNN: Deep neural network
EHR: Electronic health records
EstBB: Estonian biobank
HL7: Health level seven (international)
HGRA: Human Gene Research Act
HR: Hazard ratio
HTML: Hypertext markup language
ICD-10: International classification of diseases (revision 10)
IS: Ischemic stroke
K–M: Kaplan–Meier
KNN: *K*-Nearest neighbors algorithm
lowess: Locally weighted scatterplot smoothing
LOINC: Logical observation identifiers, names, and codes
LR: Logistic regression
ML: Machine learning
*p*: *p*-Value
RF: Random forests algorithm
SD: Standard deviation
SE: Standard error
STACC: Software Technology and Applications Competence Centre, Tartu, Estonia
UTARTU: University of Tartu, Estonia
XML: Extensible markup language
