# Supplementary material for: Common clinical blood and urine biomarkers for ischemic stroke: an Estonian Electronic Health Records database study
Source: Eur J Med Res. 2023 Mar 25;28:133. doi: 10.1186/s40001-023-01087-6 (PMC10039346; doi:10.1186/s40001-023-01087-6)
Supplement: Supplementary file 1 — Additional file 1: Table S1. Abbreviations used in the article. Table S2. Clinical parameters mentioned in the article. Text S3. Raw data extraction workflow. Text S4. Semi-automatic pipeline for cleaning raw tabular EHR data for downstream analyses of IS. Fig. S5. Sources for phenotype information from the EstBB. Text S6. Filters used in Association Rule Mining (ARM) to identify potentially interesting rules for further testing. Text S7. R script to transform numerical clinical data for Logistic Regression (LR). Text S8. R script used for Cox Proportinal Hazards (CPH) model and Kaplan-Meier (K-M) graphs. Text S9. Deep neural networks (DNN) implementation. Table S10. Five association rules (ARMs) identified. Fig. S11. Lowess curve examples. Table S12. Summary of lowess. Table S13. Summary of Logistic Regression (LR). Fig. S14. Kaplan-Meier graphs for CPs with P<0.001 and proportional hazards. Table S15. Summary of Cox Proportional Hazards (CPH) model. Fig. S16. Kaplan-Meier graphs for B.Plt. Fig. S17. Kaplan-Meier graphs for B.MCH. Fig. S18. Kaplan-Meier graphs for S.P.HDL.Chol/S.P.Chol. Fig. S19. Kaplan-Meier graphs for B.Lymph.%/B.Neut.%. Table S20. Data sources for ML models. Fig. S21. Workflow of ML model creation and testing. Table S22. The ML ensemble models tested. [file 40001_2023_1087_MOESM1_ESM.pdf]

Supplementary Materials

Index

PAGE **SUPPLEMENTARY MATERIAL**
Table S1. Abbreviations used in the article.
Table S2. Clinical parameters mentioned in the article.
Text S3. Raw data extraction workflow.
Text S4. Semi-automatic pipeline for cleaning raw tabular EHR data for downstream analyses of IS.
Fig. S5. Sources for phenotype information from the EstBB.
Text S6. Filters used in Association Rule Mining (ARM) to identify potentially interesting rules for further testing.
Text S7. R script to transform numerical clinical data for Logistic Regression (LR).
Text S8. R script used for Cox Proportinal Hazards (CPH) model and Kaplan-Meier (K-M) graphs.
Text S9. Deep neural networks (DNN) implementation.
Table S10. Five association rules (ARMs) identified.
Fig. S11. Lowess curve examples.
Table S12. Summary of lowess.
Table S13. Summary of Logistic Regression (LR).
Fig. S14. Kaplan-Meier graphs for CPs with P<0.001 and proportional hazards.
Table S15. Summary of Cox Proportional Hazards (CPH) model.
Fig. S16. Kaplan-Meier graphs for B.Plt.
Fig. S17. Kaplan-Meier graphs for B.MCH.
Fig. S18. Kaplan-Meier graphs for S.P.HDL.Chol/S.P.Chol.
Fig. S19. Kaplan-Meier graphs for B.Lymph.%/B.Neut.%.
Table S20. Data sources for ML models.
Fig. S21. Workflow of ML model creation and testing.
Table S22. The ML ensemble models tested.

Table S1. Abbreviations used in the article.

**AR**	Association Rule
**ARM**	Association Rule Mining
**ATC**	Anatomical Therapeutic Chemical (code)
**AUC**	Area Under the Curve (here same as AUROC)
**CP(s)**	Clinical Parameter(s)
**CPH**	Cox Proportional Hazards Model
**DNN**	Deep Neural Network
**EHR**	Electronic Health Records
**EstBB**	Estonian Biobank
**HL7**	Health Level Seven (international)
**HGRA**	Human Gene Research Act
**HR**	Hazard Ratio
**HTML**	Hypertext Markup Language
**ICD-10**	International Classification of Diseases (revision 10)
**IS**	Ischemic Stroke
**K-M**	Kaplan-Meier
**KNN**	K-Nearest Neighbors algorithm
**lowess**	Locally Weighted Scatterplot Smoothing
**LOINC**	Logical Observation Identifiers, Names, and Codes
**LR**	Logistic Regression
**ML**	Machine Learning
**P**	P-value
**RF**	Random Forests Algorithm
**SD**	Standard Deviation
**SE**	Standard Error
**STACC**	Software Technology and Applications Competence Centre, Tartu, Estonia
**UTartu**	University of Tartu, Estonia
**XML**	Extensible Markup Language

Table S2. Clinical parameters mentioned in the article.

Code	Name	Material
B.Hb	Hemoglobin	Full blood
B.HbA1c	Hemoglobin A1c / Hemoglobin (total)	Full blood
B.Hct	Hematocrit	Full blood
B.Lymph.#	Lymphocyte count	Full blood
B.Lymph.%	Lymphocytes / leukocytes	Full blood
B.MCHC	Erythrocyte mean corpuscular hemoglobin concentration	Full blood
B.MCHC	Erythrocyte mean corpuscular hemoglobin concentration	Full blood
B.Mono.%	Monocytes / leukocytes	Full blood
B.MPV	Platelet mean volume	Full blood
B.Neut.#	Neutrophils	Full blood
B.Neut.%	Neutrophils / leukocytes	Full blood
B.RBC	Erythrocytes	Full blood
B.RDW.CV	Erythrocyte distribution width	Full blood
B.RDW.SD	Erythrocyte distribution width	Full blood
B.Ret%	Reticulocytes / erythrocytes	Full blood
B.Segmented.Neut.%	Segmented neutrophils / leukocytes	Full blood
eGFR	Estimated glomerular filtration rate	Full blood
eGFR.CKD.EPI	Estimated gromerular filtration rate, Chronic Kidney Disease Epidemiology Collaboration (eq)	Full blood
P.PT.%	Prothrombin activity, as activity percentage	Plasma
P.APTT	Activated partial thromboplastin time	Plasma
S.P.ALAT	Alanine aminotransferase	Serum
S.P.Alb	Albumin	Serum
S.P.ALP	Alkaline phosphatase	Serum
S.P.Ca	Calcium	Serum
S.P.CA.125	Cancer antigen 125	Serum
S.P.Chol	Cholesterol in Serum	Serum
S.P.CK	Creatine kinase	Serum
S.P.Crea	Creatinine	Serum
S.P.CRP	C reactive protein	Serum
S.P.cTnT.hs	Cardiac troponin subunit T, high sensitivity assay	Serum
S.P.Fer	Ferritin	Serum
S.P.HDL.Chol	Cholesterol in HDL	Serum
S.P.LDL.Chol	Cholesterol in LDL	Serum
S.P.UA	Uric acid	Serum
S.P.Urea	Urea	Serum
U.pH.strip	Urine pH (strip)	Urine
U.Prot.strip	Urine protein (strip)	Urine

Text S3. Raw data extraction workflow.

Overview of data sources

Clinical data for this study originate from the digital Estonian Health Records (EHR). They are stored according to the HL7 V3 documentation standard [SuppRef1]. The central collection of EHR started in 2008 [SuppRef2] with a limited set of data providers and was then gradually extended to all service providers. The exact structure of the documents has been constantly evolving. The governing organization (Estonian Health and Welfare Information Systems Centre) has released 11 document standards [SuppRef3] that further specify many sub-standards for different document types. The EHR database owned by the Estonian Biobank (EstBB) is a subset of the central repository.

The main bulk of documents are stationary and ambulatory epicrisis documents with different version numbers. In our study, the most common version of ambulatory epicrisis is version 3 (152,067) and the most common version of stationary epicrisis is Version 2 (16,231). A more detailed breakdown is given in the Table 1 which shows that other document versions are also abundant.

Table 1. Number of documents in each version

Ambulatory epicrisis		Stationary epicrisis	
Version 3	152067	Version 2	16231
Version 4	121223	Version 3	13638
Version 2	31315	Version 6	2424
Version 5	27826	Version 1.1	2118
Version 7	22530	Version 5	1753
Version 8	15254	Version 8	1555
Version 6	13333	Version 7	1112
Version 9	12601	Version 1.2	1076
Version 1.1	4120		

Each standard defines a set of fields that can be filled out by the data provider. Some of them (such as the identity of the patient and healthcare provider) are mandatory but most are optional. Some of the fields can have sub-fields. For instance, the field for laboratory test results consists of individual measurements.

In the beginning of standardization, most of the data fields were unstructured. Later these fields were replaced with structured data formats. However, unstructured fields were not deprecated to preserve

compatibility between different data formats. As a result, the same data can be stored in different fields and formats. Moreover, medical personnel who use epicrises documents never see the structured data. As a result, structured data entries are often low quality or inconsistent with unstructured data. To resolve these issues, several data consolidation steps were added to the data cleaning workflow.

There is a special analysis section in epicrisis documents that describes the results of clinical laboratory tests. The section contains two subfields: one in the structured XML format and the other in more flexible HTML table format. The latter is used by medical personnel. Not all epicrises use this section to store the outcomes of clinical parameters. In older documents, the outcomes of clinical tests are stored as printout tables inside unstructured text fields. These tables are more complicated to parse than XML and HTML tables as there are no dedicated markup symbols for defining individual subfields. Finally, doctors can enter the outcomes of clinical measurements directly into medical records. The latter is commonly done for clinical parameters that can be measured in the doctors' office, such as weight, blood pressure, cholesterol and blood glucose levels.

Data extraction pipeline

For this study, we extracted 3,136,857 measurement outcomes of individual clinical parameters (CPs) from the analysis section and ignored the data in the printout tables and in-text descriptions to avoid various data quality issues. More precisely, we discarded 98,736 printout tables with approximately 900 thousand measurements (approx. 29% of extracted measurements). The actual information content is slightly lower due to data duplication between printouts and structured data. In the subsequent data analysis steps all non-numeric measurements (mostly textual descriptions) were discarded.

When medical personnel order analyses, they usually register the required tests in their institutional medical information system, and then the specimens can be collected and tested at the site, or shipped to an independent laboratory. Laboratories with clinical accreditation may provide their services as departments of the hospitals or may work independently to cover country-wide ambulatory service and/or primary care units (general practitioners). After the test results have been validated by a medical specialist (usually the same person who placed the order), Estonian law demands that the results are to be sent to the Health Information System [SuppRef4] or more specifically EHR where patients can access the results.

Therefore, EHR consolidates data from more than 20 independent laboratories [SuppRef5]. In order to handle such diversity, a special national-level LOINC task force [SuppRef6] was created in 2011 to standardize test nomenclature between laboratories. One end result of this standardisation process is the ELHR terminology database [SuppRef7] provided by the Estonian Society for Laboratory Medicine. This database contains standardized names for lab measurements, together with the corresponding LOINC codes. As the database does not cover all possible term variations used in epicrisis documents, we created our own mapping by adding variations of frequently occurring parameter names.

Our data extraction pipeline consists of three main tasks: (i) data import and basic clean-up; (ii) consolidation of different data sources; (iii) conversion to final format. The first task itself consists of four sub-steps: (a) parsing; (b) data cleansing; (c) LOINC code assignment; (d) removal of duplicate entries.

Parsing. The only source-specific step in this task is parsing. For the XML source, specific XML tags and their attributes are mapped directly to the fields of an SQL table, see the Table 2 for detailed description.

Table 2. Corresponding SQL tables

**Section in epicrisis document**	**Corresponding SQL tables**
Allergy	allergy, allergy_entry
Medical case and encounters	ambulatory_case, department_stay, hospital_stay
Diagnosis	main_diagnosis, complication_diagnosis, by_illness_diagnosis, outer_cause_diagnosis
Anamnesis and disease progression	anamnesis
Studies and procedures	procedures, procedures_entry
Clinical measurements	analysis, analysis_entry
Objective findings	objective_finding
Drug prescriptions	drug, drug_entry
Surgeries	surgery, surgery_entry, surgery_entry_method, surgery_entry_qualifier
Summary	summary
Other sections	14 other tables

For HTML source, the HTML table is first extracted from the XML document and then converted to a matrix with different entries. This process is non-trivial as an HTML table may contain cells that span over several rows and columns. The value of such a cell is copied to each matrix entry covered by the cell. After that, all matrix entries are imported to an SQL table with the same format as for the XML source. The latter requires advanced pattern matching as the HTML data comes in four different formats with subtypes that can be distinguished only based on the content of the column header. Some table formats do not

contain fields from the SQL table. This will significantly complicate the data consolidation step later.

Data cleansing. This and the following two steps are identical for both data sources. In the data cleansing step, we normalise the content of individual data fields. Firstly, data fields can contain a lot of noise: errors in font encodings, different ways to print decimal fractions and missing values, dummy values and insertions of random substrings. Secondly, we need to separate, normalise and expand different formats of measurement values: integers, decimal fractions, ratios, time series etc. To handle time series, we need to split one data entry into many entries and assign a time series identifier that allows users to recover grouping after the entire pipeline is negotiated.

LOINC assignment. Consistent LOINC code assignment with high coverage is a non-trivial task. A LOINC code is determined by six parameters, four of which are observable in the data: analyte to be measured, substance to be analysed, the scale and units of measurement. Unfortunately, measurement units are not recorded for a large proportion of measurements and analyte names have many variants depending on the laboratory and the current coding version. Moreover, the name of the analyte can occur in two different fields (analysis name and parameter name) which further complicates the LOINC assignment. To resolve these issues, we first normalised the layout of analysis and parameter names and units, then tried to identify the analysis substance and finally apply many different mappings to determine LOINC code for each row of the SQL table. Some of these are based on the ELHR terminology database and some of them are manually created. Such a setup allows us to gradually enhance the coverage and accuracy of the final LOINC assignment, focus on the relevant subset of clinical parameters and resolve errors detected by downstream data analysis projects.

Duplicate removal. The final duplicate removal step eliminates identical measurement entries in the same epicrisis document. These can occur if the same analysis block is added more than once to the epicrisis document or the same measurement is duplicated due a programming error. This step does not eliminate all measurement duplicates. For instance, doctors often extend an existing epicrisis document, which can lead to document duplication. Secondly, the same measurement can be included into different epicrisis documents.

Data consolidation. The second task in the pipeline consolidates the output of XML and HTML sources. For that we try to match

---

7

measurement entries inside the same epicrisis document. Missing values in measurement entries make this task non-trivial. Essentially, we have to decide ordering over partial matches so that the matches with the strongest evidence are preferred to more heuristic alternatives. Both data sources can specify the analysis name, parameter name, parameter unit, effective time and reference value. For the ideal match all fields must be equal but there are many other combinations that can correspond to a true match. Each of them corresponds to a certain programming error or the limitation of the HTML data source. For instance, some XML entries do not contain a parameter name or its value is a dummy value. Then the corresponding match without the parameter name allows to recover what was measured. At first glance, a valid partial match should have the same measurement value. However, in rare cases even the values are different due to the different formatting rules.

The matching procedure first pairs perfect matches and then goes through the list of partial matches until all measurement entries are matched or we are left with unpaired entries. The exact ordering of partial match steps was fixed through careful repeated exploration. The resulting workflow is the most complex and resource consuming block inside the entire pipeline.

Data normalization. The final data normalization step uses matched measurement entries to fill in missing data and/or reconcile differences in field values. When the field has different values we intentionally prefer the HTML source as this is the source medical personnel see and validate. The only reason to prefer the XML source would be the artefacts from anonymization. As anonymisation of epicrises documents is done before the data extraction and cleanup, some parts of laboratory measurements entries are censored (e.g. ELISA analysis as Elisa is a common name).

The resulting data is reshaped into the final format described in the Table 3.

Table 3. Column information

Column	Description
epi_id	document identifier
loinc_code	official loinc code
elabor_t_lyhend	official measurement name in Estonian
analysis_name	clinical test
parameter_name	analyte name
parameter_unit	measurement unit
time_series_block	identifier for measurement serie
effective_time	measurement time
value	measurement value
value_type	value type (float, ratio, text etc)
reference_values	reference value interval

In particular, note that we can separate individual measurements from a series of measurements such as 24 *h* glucose level monitoring. In later analysis steps, we added added sex, birth year, age at measurements and whether the person had experienced ischemic stroke beforehand.

Development details

The data extraction pipeline is built using various scripts in bash, perl, python and psql. Main task scheduling is done in Python using the Luigi library [SuppRef8]which provides a good way to handle dependencies between subtasks. Bash scripts are mainly used for calling out specific workflows. The data are stored in the PostgreSQL 11.4 database. Perl is used only in XML parsing due to legacy reasons. The corresponding XML::Simple library [SuppRef9] is outdated and one should use the modern Pyhton lxml library instead [SuppRef10]. For efficiency reasons, we implemented most data transformations directly with SQL using PLSQL functions. Most SQL scripts were embedded into Python using the psycopg2 library [SuppRef11]. HTML parsing was built on top of the Python lxml and pandas libraries [SuppRef12] and the result was imported back into the PostgreSQL database. Hard-core natural language processing and fact extraction that is built on top of the EstNLTK python library [SuppRef13] which uses PostgreSQL database JSON capabilities to serialise textual annotations. We mostly use rule-based methods for text segmentation and fact extraction. This was optimal as most text fields are semi-structured – contain well-defined headers and table printouts have a regular form. Even the facts about medical procedures, medications and clinical measurements are mostly

regular although the variation makes it much harder to derive good rules.

To get consistent improvement in data extraction quality, we used several data validation scripts to catch obvious errors and monitor the quality of LOINC assignment and source consolidation. We did not perform any manual curation or statistical validation of extracted measurements. In particular, we did not search for obvious outliers and multimodality in histograms. The latter is a strong indicator that values of a lab measurement are stored in different units. These steps were carried out in the actual data analysis.

The total amount of work has been staggering. It has taken well over 10 man-years of development time to establish the entire platform for data extraction including large updates into the EstNLTK library. Most of this has been laying the groundwork. With the current knowledge it would be possible to replicate the workflow for clinical parameter extraction with 2-3 man-years. The bulk of the work would still go into harmonization of different data formats, assembly and curation of various standardization mappings.

Resource consumption

The workflow does not require specific hardware. It runs on a hardened Linux installation with standard hardware – no GPU acceleration. It takes approximately one day to perform the entire data processing for 440181 epicrisis documents.

References

SuppRef1. Health Level Seven International. *HL7 Standards Product Brief - HL7 Version 3 Product Suite | HL7 International* Available at: https://www.hl7.org/implement/standards/product_brief.cfm?product_id=186. (Accessed: 16th January 2022)

SuppRef2. Tervise infosüsteemi edastatavate dokumentide andmekoosseisud ning nende säilitamise tingimused ja kord. *Tervise infosüsteemi edastatavate dokumentide andmekoosseisud ning nende säilitamise tingimused ja kord–Riigi Teataja* Available at: https://www.riigiteataja.ee/akt/13029628. (Accessed: 16th January 2022)

SuppRef3. Tervise ja heaolu infosüsteemide keskus. Available at: http://pub.e-tervis.ee/standards2/Standards. (Accessed: 16th January 2022)

SuppRef4. Tervise infosüsteem. *TEHIK* Available at: https://www.tehik.ee/tervise-infosusteem. (Accessed: 16th January 2022)

SuppRef5. Health Record - e-Estonia. *e* (2021). Available at: https://e-estonia.com/solutions/healthcare/e-health-records/. (Accessed: 16th January 2022)

SuppRef6. Estonian Society for Laboratory Medicine. *Eesti Laborimeditsiini Ühing* (2019). Available at: https://www.elmy.ee/estonian-society-for-laboratory-medicine/. (Accessed: 16th January 2022)

SuppRef7. *ELHR* Available at: https://elhr.digilugu.ee/data/algandmedList.html. (Accessed: 16th January 2022)

SuppRef8. https://github.com/spotify/luigi/

SuppRef9. XML::Simple. *MetaCPAN icon* Available at: https://metacpan.org/pod/XML::Simple. (Accessed: 16th January 2022)

SuppRef10. XML and HTML with Python. *lxml* Available at: https://lxml.de/. (Accessed: 16th January 2022)

SuppRef11. *Psycopg* Available at: https://www.psycopg.org/. (Accessed: 16th January 2022)

SuppRef12. *Pandas* Available at: https://pandas.pydata.org/. (Accessed: 16th January 2022)

SuppRef13. Laur, S., Orasmaa, S., Särg, D. & Tammo, P. EstNLTK 1.6: Remastered Estonian NLP Pipeline. *ACL Anthology* Available at: https://aclanthology.org/2020.lrec-1.884/. (Accessed: 16th January 2022)

Text S4. Semi-automatic pipeline for cleaning raw tabular EHR data for downstream analyses of IS.

1. Remove non-ASCII characters and replace with "_".

2. Remove entries with missing or non-positive concentration values.

3. Additional LOINC import.

If LOINC is missing then search for it in free text columns.

4. Fix and format measurement dates.

5. Validate potential cases and controls (retrieved from EHR) against the EstBB database.

6. Divide combinatorial LOINCs (representing several parameters) into single-parameter LOINCs.

7. Remove duplicate measurements from the same day (retain the first measurement).

8. Add additional fields of information such as sex, age etc.

9. Remove measurements that occur after the first IS incidence.

10. Filter out LOINCs that occur less than 45 times among the cases combined.

11. Create the connection between LOINC and human readable description.

12. Filter out individuals who had less than 10 reported measurements.

13. Filter out control individuals with certain ICD-10 codes (I60, I61, I62, I64) and ATC codes (B01, M01A, M01BA03, N02BA).

14. Generate histograms for all LOINCs to detect those with abnormal distributions.

15. Fix the abnormally distributed LOINCs:

a) If two separate distributions: divide into two new LOINCs

b) If overlapping distributions: separate into two LOINCs and remove overlapping samples

c) If no good solution: remove the corresponding LOINC

16. Standardize the concentration values: value = ln(1+value)

Remove outliers (3 stdev)

17. Match 3 controls (from among 7398) to each case (950)

18. Adjust values (each LOINC separately) for sex and age.

Represent the residuals of the linear model as Z-scores.

Fig. S5. Sources for phenotype information from the EstBB.

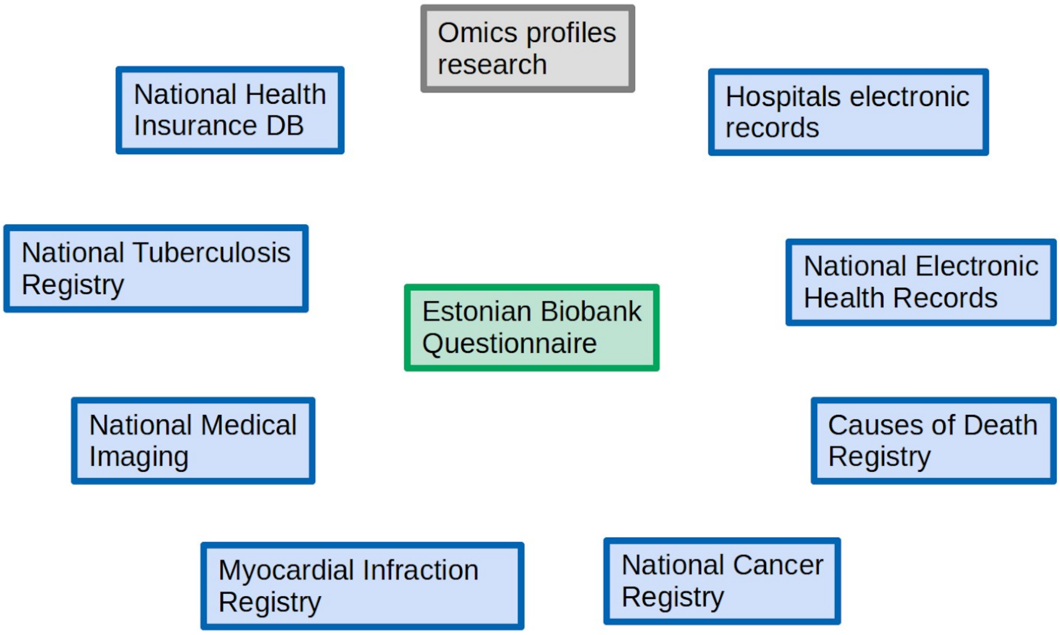

The national health databases that are linked are in blue boxes.

Text S6. Filters used in Association Rule Mining (ARM) to identify potentially interesting rules for further testing.

Confidence > 0.4
Support(case) > 10
Support(case) / max_support(control; i=20) > 1.45
Support(case) / mean_support(control; i=20) > 5
Min required control iterations with non-NA return > 1

Text S7. R script to transform numerical clinical data for Logistic Regression (LR).

```
tx <- original_values
tx[(!is.na(tx))&(abs(scale(tx))>10)]<-NA
if(max(tx,na.rm=T)<0.1) tx<-tx/max(tx,na.rm=T)
if(min(tx,na.rm=T)<0.1) tx<-tx+0.1
scale(log(tx))
```

Text S8. R script used for Cox Proportinal Hazards (CPH) model and Kaplan-Meier (K-M) graphs.

```
library(survival)
library(survminer)
library(dplyr)

data<-read.table("input.txt",header=T,strings=F)
surv_object <- Surv(time = data$futime, event = data$fustat)
fit1 <- survfit(surv_object ~ TRAIT, data = data)

png(filename = "TRAIT_1.png", width = 800, height = 800, units = "px",
pointsize = 12, bg = "white", res = NA)
ggsurvplot(fit1, data = data, pval = TRUE, subtitle = "TRAIT",
font.subtitle = c(26, "bold.italic", "black"), font.caption = c(44, "plain",
"orange"))
dev.off()

png(filename = "TRAIT_2.png", width = 800, height = 800, units = "px",
pointsize = 12, bg = "white", res = NA)
fit.coxph <- coxph(surv_object ~ TRAIT, data = data)
ggforest(fit.coxph, data = data, fontsize = 1.5)
dev.off()
```

Text S9. Deep neural networks (DNN) implementation.

Hyper parameters tested in grid search

Random forest

- max_features∈{sqrt,25,50}
- maxdepth∈{10,20,30,40,50,60}
- min samples leaf ∈ {2, 5, 8, 11}
- number of estimators ∈ {600, 1000, 1400, 1800}

FastAI tabular

- learning rate ∈ {0.005,0.01,0.02,0.025,0.05}
- number of layers ∈ {2, 3}
- number of nodes ∈ {100, 300, 500, 1000}

TabNet

- learning rate ∈ {0.005, 0.01, 0.02, 0.025}
- N_{steps} ∈ {3,4,5,6,7,8,9,10}
- $N_d = N_a$ ∈ {8,16,32,64,128}
- γ ∈ {1.0, 1.2, 1.5, 2.0}
- B ∈ {256, 512, 1924, 2048}

FastAI tabular implementation

```
  dls = TabularDataLoaders.from_df(cv_dataset, y_names="I63_CASE",
   y_block = CategoryBlock,
   cat_names = cat_names,
   cont_names = cont_names,
   procs = procs)

   learn = tabular_learner(dls, layers=[500 , 1000], metrics=roc_auc,
opt_func=Adam)
```

Definition of FastAI tabular model in code, using FastAI library.

The cat_names is a list of all categorical variables (sex, ICD-10), cont_names is a list of all continuous variables (all others). The procs are procedures done, in this case the FillMissing and Categorify are called.

The detailed descriptions of the functions are given in the FastAI documentation found https://docs.fast.ai/.

Detailed model summary for the DNN created by the FastAI tabular

```
TabularModel(
  (embeds): ModuleList(
    (0): Embedding(3, 3)
    (1): Embedding(446, 49)
  )
  (emb_drop): Dropout(p=0.0, inplace=False)
  (bn_cont): BatchNorm1d(149, eps=1e-05, momentum=0.1, affine=True, track_running_stats=True)
  (layers): Sequential(
    (0): LinBnDrop(
      (0): Linear(in_features=201, out_features=500, bias=False)
      (1): ReLU(inplace=True)
      (2): BatchNorm1d(500, eps=1e-05, momentum=0.1, affine=True, track_running_stats=True)
    )
    (1): LinBnDrop(
      (0): Linear(in_features=500, out_features=1000, bias=False)
      (1): ReLU(inplace=True)
      (2): BatchNorm1d(1000, eps=1e-05, momentum=0.1, affine=True, track_running_stats=True)
    )
    (2): LinBnDrop(
      (0): Linear(in_features=1000, out_features=2, bias=True)
    )
  )
)
```

FastAI implementation of TabNet

```
  dls = TabularDataLoaders.from_df(cv_dataset, y_names="I63_CASE",
   y_block = CategoryBlock,
   cat_names = cat_names,
   cont_names = cont_names,
   procs = procs, bs = 2048)
   splits = RandomSplitter(valid_pct=0.1)(range_of(cv_dataset))
   to = TabularPandas(cv_dataset, procs, cat_names, cont_names,
y_names="I63_CASE", y_block = CategoryBlock(), splits=splits)
   emb_szs = get_emb_sz(to)
   model = TabNetModel(emb_szs, len(to.cont_names), dls.c, n_d=128,
n_a=128, n_steps=3, mask_type='entmax', virtual_batch_size=256,
gamma=1)
```

The following adaption of TabNet was used:

https://github.com/mgrankin/fast_tabnet .

Detailed model summary for the DNN created by the FastAI TabNet

TabNetModel(

(embeds): ModuleList(

(0): Embedding(3, 3)

(1): Embedding(427, 48)

)

(emb_drop): Dropout(p=0.0, inplace=False)

(bn_cont): BatchNorm1d(149, eps=1e-05, momentum=0.1, affine=True, track_running_stats=True)

(tab_net): TabNetNoEmbeddings(

(initial_bn): BatchNorm1d(200, eps=1e-05, momentum=0.01, affine=True, track_running_stats=True)

(encoder): TabNetEncoder(

(initial_bn): BatchNorm1d(200, eps=1e-05, momentum=0.01, affine=True, track_running_stats=True)

```
  (initial_splitter): FeatTransformer(
    (shared): GLU_Block(
      (shared_layers): ModuleList(
        (0): Linear(in_features=200, out_features=512, bias=False)
        (1): Linear(in_features=256, out_features=512, bias=False)
      )
      (glu_layers): ModuleList(
        (0): GLU_Layer(
          (fc): Linear(in_features=200, out_features=512, bias=False)
          (bn): GBN(
            (bn): BatchNorm1d(512, eps=1e-05, momentum=0.02, affine=True, track_running_stats=True)
          )
        )
        (1): GLU_Layer(
          (fc): Linear(in_features=256, out_features=512, bias=False)
          (bn): GBN(
            (bn): BatchNorm1d(512, eps=1e-05, momentum=0.02, affine=True, track_running_stats=True)
          )
        )
      )
    )
    (specifics): GLU_Block(
      (glu_layers): ModuleList(
        (0): GLU_Layer(
          (fc): Linear(in_features=256, out_features=512, bias=False)
          (bn): GBN(
            (bn): BatchNorm1d(512, eps=1e-05, momentum=0.02, affine=True, track_running_stats=True)
          )
```

```
            )
            (1): GLU_Layer(
              (fc): Linear(in_features=256, out_features=512, bias=False)
              (bn): GBN(
                (bn): BatchNorm1d(512, eps=1e-05, momentum=0.02, affine=True, track_running_stats=True)
              )
            )
          )
        )
      )
      (feat_transformers): ModuleList(
        (0): FeatTransformer(
          (shared): GLU_Block(
            (shared_layers): ModuleList(
              (0): Linear(in_features=200, out_features=512, bias=False)
              (1): Linear(in_features=256, out_features=512, bias=False)
            )
            (glu_layers): ModuleList(
              (0): GLU_Layer(
                (fc): Linear(in_features=200, out_features=512, bias=False)
                (bn): GBN(
                  (bn): BatchNorm1d(512, eps=1e-05, momentum=0.02, affine=True, track_running_stats=True)
                )
              )
              (1): GLU_Layer(
                (fc): Linear(in_features=256, out_features=512, bias=False)
                (bn): GBN(
                  (bn): BatchNorm1d(512, eps=1e-05, momentum=0.02, affine=True, track_running_stats=True)
```

```
        )
      )
    )
  )
  (specifics): GLU_Block(
    (glu_layers): ModuleList(
      (0): GLU_Layer(
        (fc): Linear(in_features=256, out_features=512, bias=False)
        (bn): GBN(
          (bn): BatchNorm1d(512, eps=1e-05, momentum=0.02, affine=True,
track_running_stats=True)
        )
      )
      (1): GLU_Layer(
        (fc): Linear(in_features=256, out_features=512, bias=False)
        (bn): GBN(
          (bn): BatchNorm1d(512, eps=1e-05, momentum=0.02, affine=True,
track_running_stats=True)
        )
      )
    )
  )
)
(1): FeatTransformer(
  (shared): GLU_Block(
    (shared_layers): ModuleList(
      (0): Linear(in_features=200, out_features=512, bias=False)
      (1): Linear(in_features=256, out_features=512, bias=False)
    )
    (glu_layers): ModuleList(
      (0): GLU_Layer(
```

```
      (fc): Linear(in_features=200, out_features=512, bias=False)
      (bn): GBN(
        (bn): BatchNorm1d(512, eps=1e-05, momentum=0.02, affine=True,
track_running_stats=True)
      )
    )
    (1): GLU_Layer(
      (fc): Linear(in_features=256, out_features=512, bias=False)
      (bn): GBN(
        (bn): BatchNorm1d(512, eps=1e-05, momentum=0.02, affine=True,
track_running_stats=True)
      )
    )
  )
)
(specifics): GLU_Block(
  (glu_layers): ModuleList(
    (0): GLU_Layer(
      (fc): Linear(in_features=256, out_features=512, bias=False)
      (bn): GBN(
        (bn): BatchNorm1d(512, eps=1e-05, momentum=0.02, affine=True,
track_running_stats=True)
      )
    )
    (1): GLU_Layer(
      (fc): Linear(in_features=256, out_features=512, bias=False)
      (bn): GBN(
        (bn): BatchNorm1d(512, eps=1e-05, momentum=0.02, affine=True,
track_running_stats=True)
      )
    )
```

```
        )
      )
    )
    (2): FeatTransformer(
      (shared): GLU_Block(
        (shared_layers): ModuleList(
          (0): Linear(in_features=200, out_features=512, bias=False)
          (1): Linear(in_features=256, out_features=512, bias=False)
        )
        (glu_layers): ModuleList(
          (0): GLU_Layer(
            (fc): Linear(in_features=200, out_features=512, bias=False)
            (bn): GBN(
              (bn): BatchNorm1d(512, eps=1e-05, momentum=0.02, affine=True, track_running_stats=True)
            )
          )
          (1): GLU_Layer(
            (fc): Linear(in_features=256, out_features=512, bias=False)
            (bn): GBN(
              (bn): BatchNorm1d(512, eps=1e-05, momentum=0.02, affine=True, track_running_stats=True)
            )
          )
        )
      )
      (specifics): GLU_Block(
        (glu_layers): ModuleList(
          (0): GLU_Layer(
            (fc): Linear(in_features=256, out_features=512, bias=False)
            (bn): GBN(
```

```
          (bn): BatchNorm1d(512, eps=1e-05, momentum=0.02, affine=True, track_running_stats=True)
        )
      )
      (1): GLU_Layer(
        (fc): Linear(in_features=256, out_features=512, bias=False)
        (bn): GBN(
          (bn): BatchNorm1d(512, eps=1e-05, momentum=0.02, affine=True, track_running_stats=True)
        )
      )
    )
  )
 )
)
(att_transformers): ModuleList(
  (0): AttentiveTransformer(
    (fc): Linear(in_features=128, out_features=200, bias=False)
    (bn): GBN(
      (bn): BatchNorm1d(200, eps=1e-05, momentum=0.02, affine=True, track_running_stats=True)
    )
    (selector): Entmax15()
  )
  (1): AttentiveTransformer(
    (fc): Linear(in_features=128, out_features=200, bias=False)
    (bn): GBN(
      (bn): BatchNorm1d(200, eps=1e-05, momentum=0.02, affine=True, track_running_stats=True)
    )
    (selector): Entmax15()
```

```
        )
        (2): AttentiveTransformer(
          (fc): Linear(in_features=128, out_features=200, bias=False)
          (bn): GBN(
                  (bn): BatchNorm1d(200, eps=1e-05, momentum=0.02, affine=True, track_running_stats=True)
          )
          (selector): Entmax15()
        )
      )
    )
    (final_mapping): Linear(in_features=128, out_features=2, bias=False)
  )
)
```

Table S10. Five association rules (ARMs) identified.

A	Pearson	ALL	MEN	WOMEN	YOUNG	OLD
B.Lymph.%_LOW + B.Neut.%_HIGH	-0.86	61; 6.6	25; 8.8	37; 22.4	14; 7	50; 7.9
B.MCV_LOW + B.MCH_LOW	0.86	94; 11.5	2; inf	55; 4.3	15; inf	74; inf
B.Pct_HIGH + B.Plt_HIGH	0.75	49; 10.8	20; 1.66	28; 43.1	11;2.9	37; 7
S.P.UA_HIGH + S.P.Crea_HIGH	0.21	NA	13;inf	44; 5.4	NA	63; 7.3
B.Hct_LOW + B.Hb_LOW	0.77	26; inf	11; inf	14; inf	4; inf	21; inf

B	ALL	MEN	WOMEN	YOUNG	OLD
B.Lymph.%_LOW (sup(case) / sup(mean_controls))	1.57	1.24	1.77	1.65	1.57
B.Neut.%_HIGH (sup(case) / sup(mean_controls))	1.95	1.74	2.12	1.93	1.88
predicted	3.06	2.16	3.75	3.18	2.95
observed	6.6	8.8	22.4	7	7.9
observed/predicted	2.16	4.07	5.97	2.2	2.68

A: Five association rules identified. Table shows Pearson correlation between the individual items and **a)** support (case), **b)** support(case) / mean_support (controls), separated by semicolon, for the five sub-groups. **B:** Validation of the B.Lymph.%_LOW---B.Neut.%_HIGH association rule. Support (case) / support (mean_controls) is computed for both items of the rule for all study groups. Observed vs. predicted is calculated to show 2.2 – 5.97 fold enrichment.

Fig. S11. Lowess curve examples.

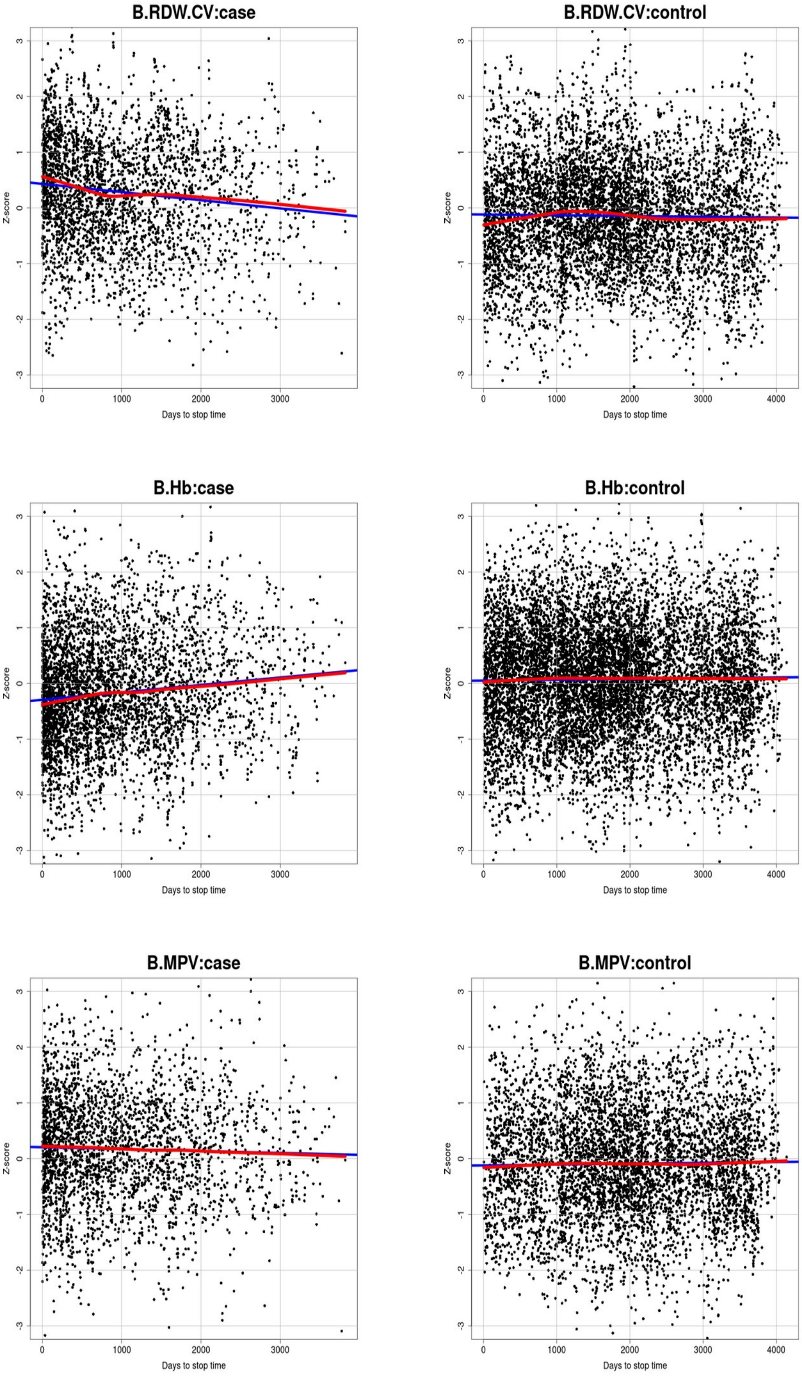

Three types of lowess curves for cases and their corresponding controls indicating an association with IS. **B.RDW.CV**: positive correlation with approaching IS only among cases, **B.Hb**: negative correlation with approaching IS only among cases, **B.MPV**: Z-scores positive for cases but negative for controls. Time zero is the time of IS (stop time). The x-axis values indicate the number of days to IS (for controls this value is estimated via the cases they are matched to). Red line is lowess, blue line is linear regression.

Table S12. Summary of lowess.

	ALL	MEN	WOMEN	YOUNG
**B.RDW.CV**	**3000**	**3000**	**3500**	**3500**
**B.MPV**	**3500**	**3500**	**3500**	1000
**B.Neut.#**	**3500**	500	**3500**	3000
**B.Hct**	**2000**	**3500**	**2000**	3500
**S.P.Chol**	**3000**	**3500**	**2500**	NA
**B.Hb**	**2000**	**2000**	**2000**	**4000**
**B.Lymph.%**	**2500**	**1500**	**2500**	**3500**
**S.P.Crea**	**3000**	**3000**	**2500**	NA
**S.P.LDL.Chol**	**2500**	3500	**2000**	500
**S.P.HDL.Chol**	2500	2500	2500	750
**S.P.Urea**	**2000**	**2000**	**2000**	1500
**B.RDW.SD**	**2000**	750	1750	750
**B.Neut.%**	2000	500	2500	500
**S.P.ALAT**	**1500**	1500	1000	NA
**S.P.ALP**	500	500	3000	NA
**S.P.UA**	1500	**2000**	NA	NA
**B.Segmented.Neut.%**	2000	NA	1500	NA
**S.P.cTnT.hs**	**1000**	400	**750**	NA
**S.P.CA.125**	1500	NA	1500	NA
**B.RBC**	1000	1000	500	NA
**S.P.Alb**	1000	NA	**1500**	NA
**P.APTT**	750	NA	1000	NA
**B.HbA1c**	750	NA	500	300
**S.P.Fer**	750	500	500	NA
**S.P.CRP**	**500**	500	**500**	NA
**eGFR**	500	NA	500	NA
**S.P.CK**	500	500	NA	NA
**S.P.HDL.Chol/S.P.Chol**	NA	2000	NA	NA
**S.P.LDL.Chol/S.P.Chol**	NA	NA	NA	NA
**B.Lymph.%/B.Neut.%**	2000	1500	1000	NA

Summary of lowess in order of total importance across all study groups. The numbers indicate trend length in days. Pink highlight = positive association with IS, blue highlight = negative association with IS. Bold numbers indicate corresponding higher than average certainty of assigning the trend as significant.

Table S13. Summary of Logistic Regression (LR).

	ALL			MEN			WOMEN			YOUNG			OLD		
	coef	se	P	coef	se	P	coef	se	P	coef	se	P	coef	se	P
B.RDW.SD	0.068	0.009	3.26E-14	0.053	0.014	1.22E-04	0.079	0.012	4.91E-11	0.104	0.029	3.96E-04	0.064	0.010	1.51E-11
B.Lymph.#	0.129	0.021	3.76E-10	0.098	0.027	2.15E-04	0.165	0.038	1.30E-05	0.133	0.055	1.67E-02	0.128	0.022	6.99E-09
B.RDW.CV	0.138	0.025	4.54E-08	0.095	0.038	1.24E-02	0.172	0.034	4.97E-07	0.206	0.083	1.29E-02	0.130	0.027	1.13E-06
B.Mono.%	-0.088	0.017	5.39E-07	-0.074	0.024	2.24E-03	-0.103	0.025	4.86E-05	-0.059	0.043	1.72E-01	-0.093	0.019	1.21E-06
S.P.HDL.Chol	-0.898	0.189	2.11E-06	-1.267	0.309	4.23E-05	-0.668	0.242	5.74E-03	-1.215	0.487	1.26E-02	-0.834	0.208	6.25E-05
P.PT.%	-0.025	0.006	1.03E-05	-0.018	0.009	4.32E-02	-0.029	0.007	8.04E-05	-0.015	0.017	3.93E-01	-0.026	0.006	1.91E-05
B.MCHC	-1.085	0.254	2.02E-05	-1.653	0.617	7.37E-03	-0.873	0.290	2.61E-03	-1.465	0.836	7.98E-02	-1.077	0.280	1.21E-04
B.Ret.%	0.625	0.173	2.95E-04	0.640	0.261	1.40E-02	0.619	0.233	7.85E-03	2.152	8E+3	1.00E+00	0.595	0.168	3.95E-04
B.Hb	-0.008	0.003	8.82E-04	-0.010	0.004	4.53E-03	-0.007	0.004	5.74E-02	-0.026	0.007	3.04E-04	-0.006	0.003	3.07E-02
XXX.Monocytes	0.122	0.038	1.30E-03	0.224	0.063	3.80E-04	0.042	0.047	3.79E-01	0.455	0.264	8.46E-02	0.113	0.038	3.20E-03
B.Neut.%	0.014	0.004	6.03E-04	0.012	0.006	5.06E-02	0.016	0.006	3.52E-03	0.018	0.010	8.34E-02	0.014	0.005	2.55E-03
B.Mono.#	-0.679	0.211	1.25E-03	-0.952	0.320	2.90E-03	-0.460	0.279	1.00E-01	-0.171	0.488	7.26E-01	-0.797	0.234	6.58E-04
U.pH.strip	-0.378	0.117	1.26E-03	-0.573	0.195	3.34E-03	-0.248	0.147	9.22E-02	-0.369	0.302	2.21E-01	-0.380	0.128	2.98E-03
S.P.Crea	0.003	0.001	1.44E-03	0.002	0.001	1.54E-01	0.004	0.001	3.08E-03	0.015	0.005	5.26E-03	0.002	0.001	6.27E-02
S.P.K	0.293	0.093	1.57E-03	0.319	0.150	3.39E-02	0.267	0.118	2.34E-02	0.456	0.266	8.59E-02	0.267	0.099	6.96E-03
S.P.HDL.Chol / S.P.Chol	-2.785	0.835	1.00E-03	-4.121	1.277	1.00E-03	-1.802	1.126	1.10E-01	-4.41	2.29	5.40E-02	-2.646	0.906	3.00E-03
S.P.LDL.Chol / S.P.Chol	-0.395	0.663	5.51E-01	0.447	0.917	6.26E-01	-1.327	0.966	1.69E-01	-2.413	1.737	1.65E-01	-0.022	0.721	9.76E-01
B.Lymph.% / B.Neut.%	-0.716	0.192	2.00E-04	-0.691	0.295	1.90E-02	-0.727	0.251	4.00E-03	-0.852	0.445	5.50E-02	-0.687	0.213	1.00E-03

Logistic regression for single CPs and three CP ratios for 1000 days before IS. P = p-value, se = standard error, coef = regression coefficient. P-values are color coded: yellow = passed Bonferroni filtering, green = nominally significant. The coef values with absolute values over 0.5 and corresponding to yellow-highlighted p-values are highlighted in dark gray.

Fig. S14. Kaplan-Meier graphs for CPs with P<0.001 and proportional hazards.

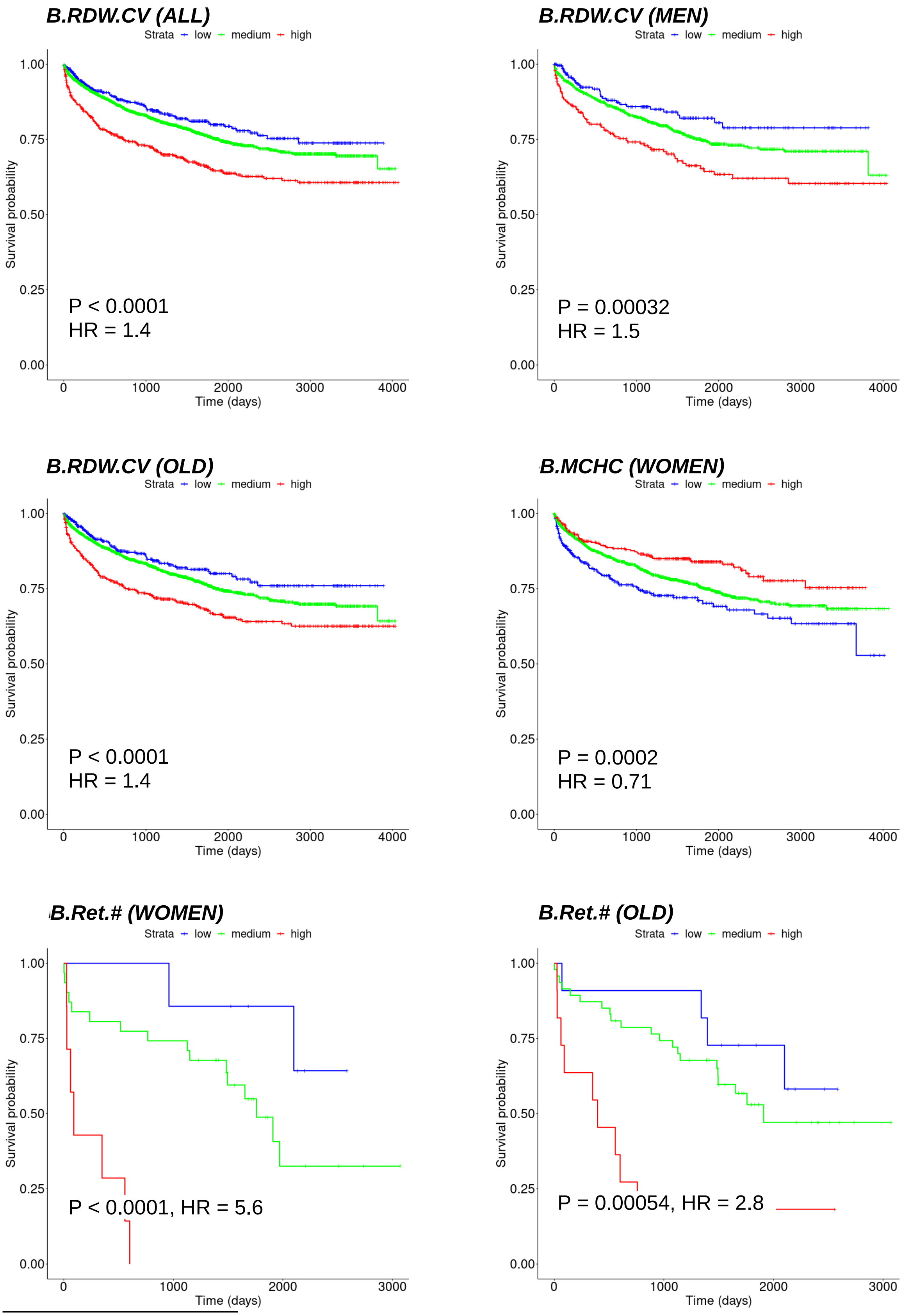

Table S15. Summary of Cox Proportional Hazards (CPH) model.

	ALL		MEN		WOMEN		YOUNG		OLD	
	P	HR	P	HR	P	HR	P	HR	P	HR
B.RDW.CV	< 1.00E-04	1.4	3.20E-04	1.5	< 1.00E-04	1.4	1.00E-01	1.3	< 1.00E-04	1.4
B.MCH	< 1.00E-04	0.97	3.40E-04	0.89	9.30E-03	1	7.80E-01	1.1	< 1.00E-04	0.96
B.Plt	< 1.00E-04	1	3.20E-02	0.96	8.30E-04	1	< 1.00E-04	0.81	1.20E-03	1
B.Ret.#	1.60E-04	2.4	6.90E-02	0.84	< 1.00E-04	5.6	NA	NA	5.40E-04	2.8
S.P.Chol	3.60E-04	0.77	< 1.00E-04	0.67	9.80E-02	0.81	6.20E-03	0.78	1.10E-03	0.75
P.PT.INR	7.50E-04	1.5	3.90E-01	1.2	< 1.00E-04	1.74	< 1.00E-04	2.9	1.60E-04	1.31
S.P.ASAT	4.00E-03	0.9	2.90E-01	0.97	2.00E-01	0.9	3.10E-01	1.1	< 1.00E-04	0.73
B.MCHC	1.30E-03	0.82	1.20E-01	1	2.00E-04	0.71	2.50E-01	0.78	3.60E-03	0.84
S.P.HDL.Chol	2.20E-04	0.75	1.70E-02	0.71	1.10E-01	0.79	1.70E-02	0.63	8.60E-02	0.83
S.P.LDH	4.10E-02	1.2	7.80E-01	0.89	4.00E-04	1.5	6.80E-01	1.2	1.90E-01	1.2
S.P.Crea	1.60E-03	1.2	5.70E-02	1.2	2.90E-02	1.2	1.40E-01	1.15	2.00E-02	1.1
S.P.LDL.Chol	2.00E-03	0.73	3.10E-03	0.7	3.20E-02	0.75	3.20E-02	0.77	1.20E-02	0.76
P.D.Di	2.20E-03	1.4	4.90E-01	1.2	5.80E-02	1.4	4.20E-01	0.58	3.40E-02	1.3
B.Hb	3.30E-03	0.85	8.00E-03	0.8	1.20E-01	0.9	2.20E-03	0.62	1.60E-01	0.89
S.P.Urea	7.20E-03	1.1	2.40E-03	1.1	1.20E-01	1	6.20E-02	1.2	1.30E-01	0.97
S.P.HDL.Chol / S.P.Chol	1.60E-02	0.84	1.00E-02	0.77	8.80E-01	0.96	< 1.00E-04	0.6	2.20E-01	0.89
S.P.LDL.Chol / S.P.Chol	7.90E-02	0.89	2.40E-01	0.88	3.10E-01	0.9	2.80E-01	1	1.20E-01	0.89
B.Lymph.% / B.Neut.%	< 1.00E-04	0.78	< 1.00E-04	0.76	< 1.00E-04	0.81	< 1.00E-04	0.59	< 1.00E-04	0.77

Top single CP hits based on the CPH sorted by minimal P-value and three CP ratios tested by CPH. P-values are color coded: red = lowest, yellow = passed Bonferroni filtering, green = nominally significant. The Hazard Ratio (HR) values representing proportional hazards are highlighted in dark gray, the rest are grayed out.

Fig. S16. Kaplan-Meier graphs for B.Plt.

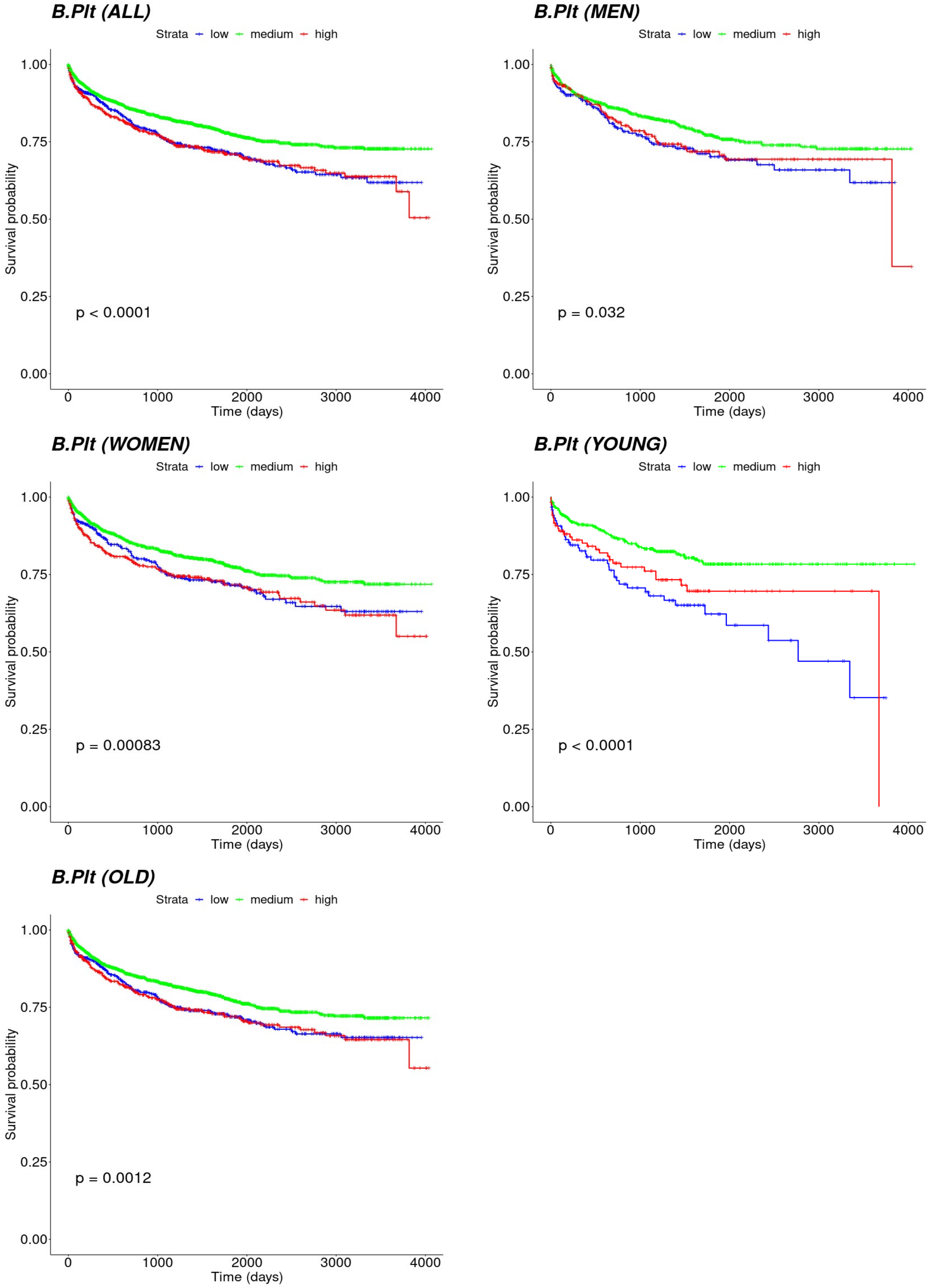

Fig. S17. Kaplan-Meier graphs for B.MCH.

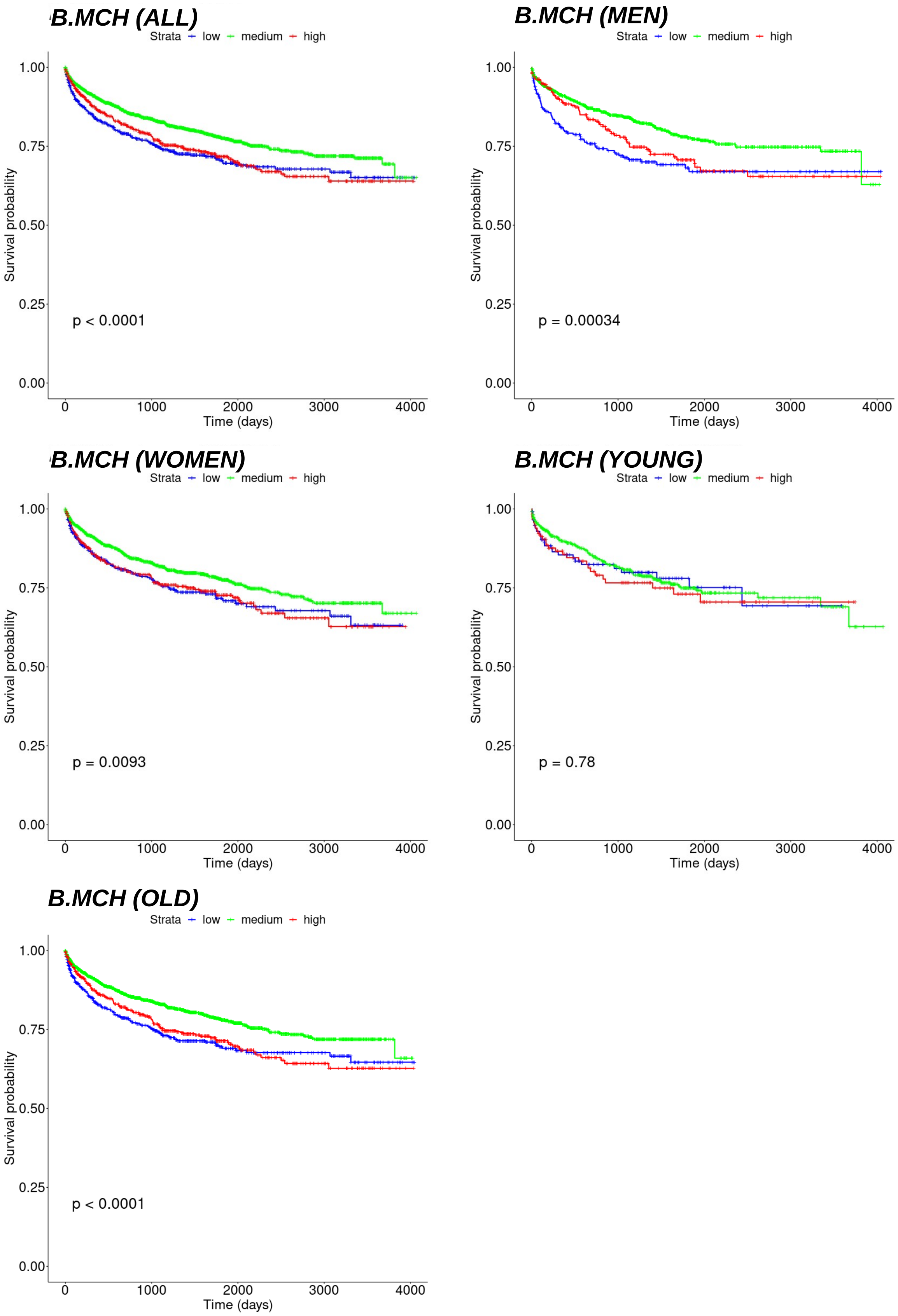

Fig. S18. Kaplan-Meier graphs for S.P.HDL.Chol/S.P.Chol.

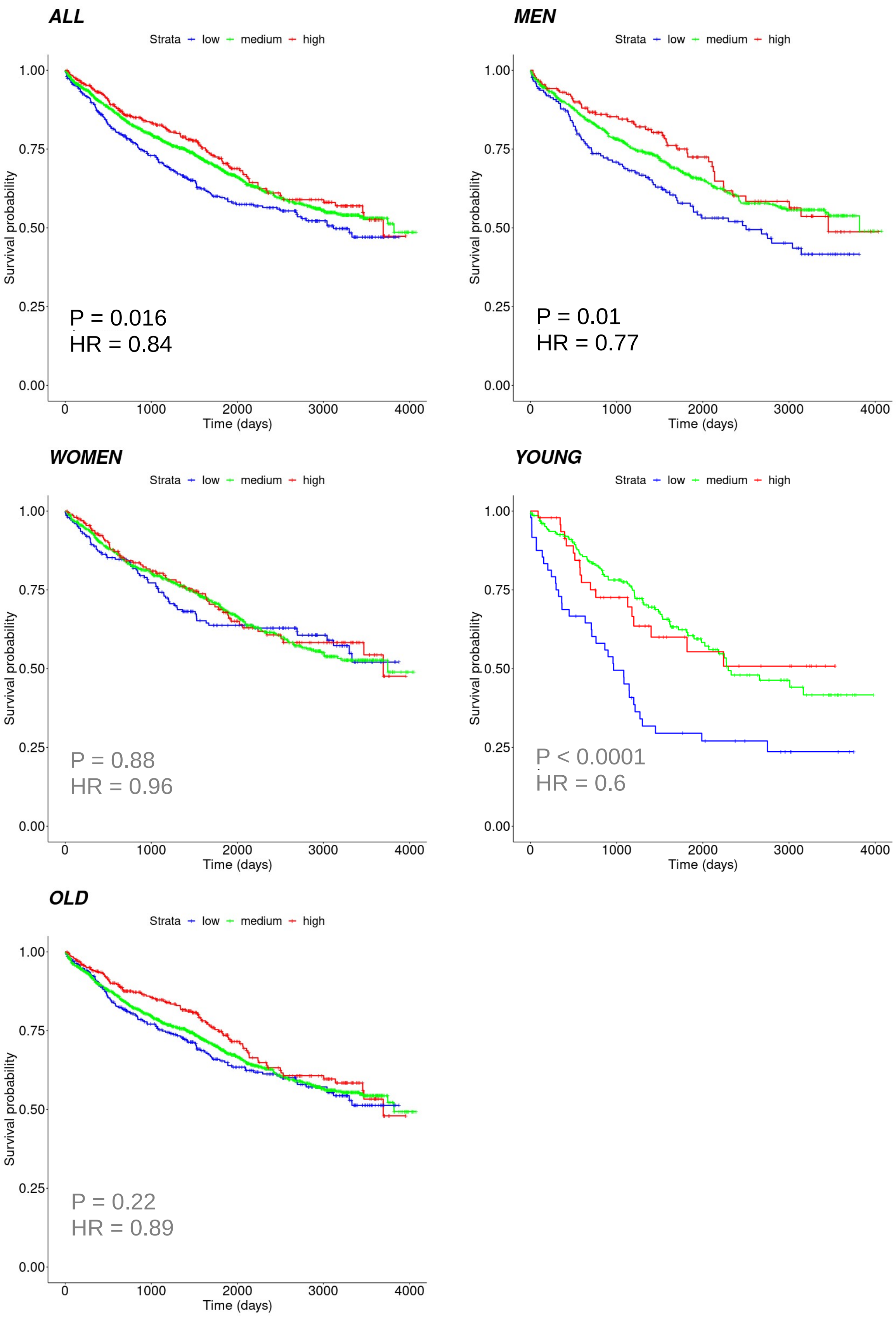

Fig. S19. Kaplan-Meier graphs for B.Lymph.%/B.Neut.%.

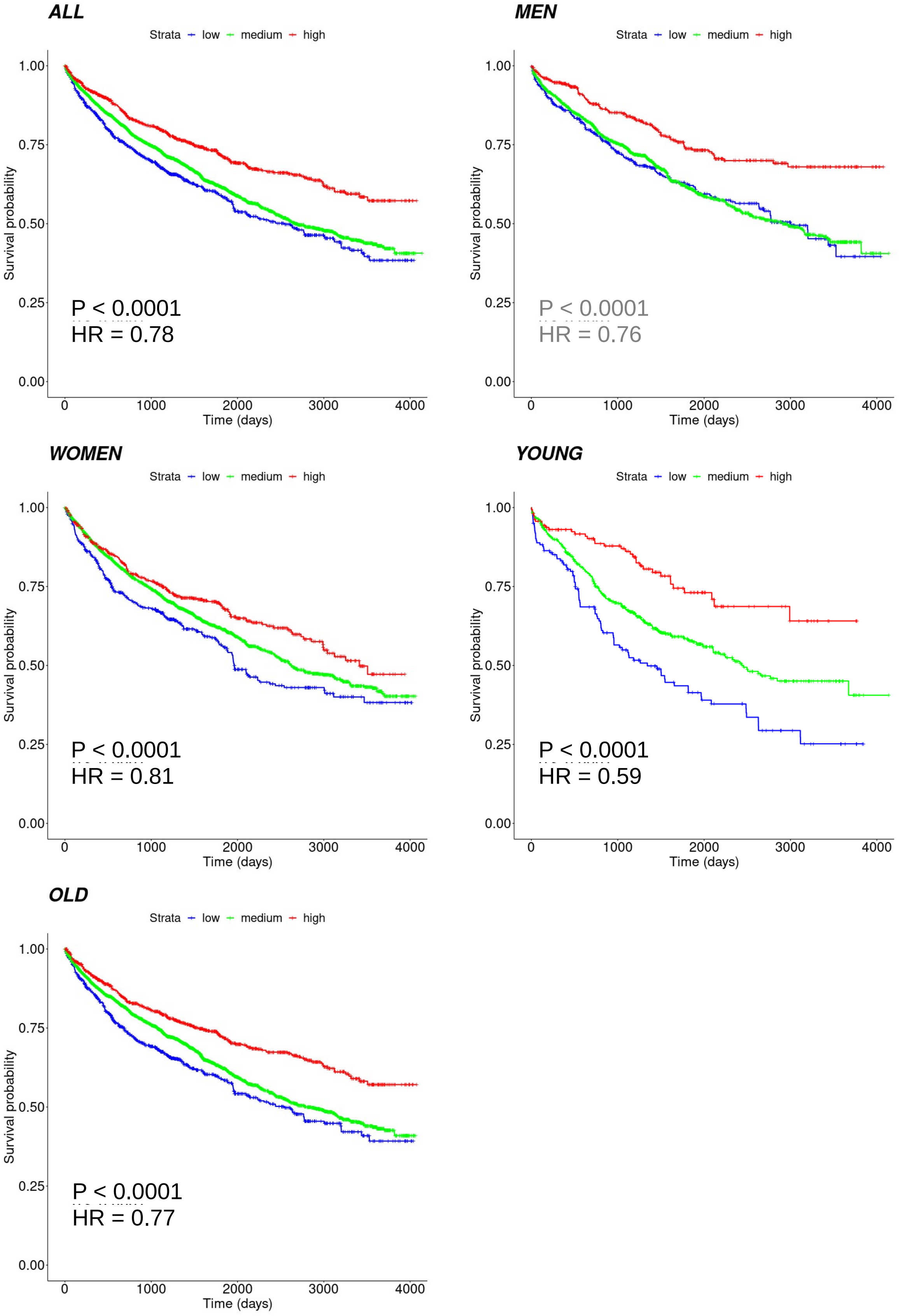

Table S20. Data sources for ML models.

Variable	Data source	Feature name	Description
Target variable	EHRs database epicrisis documents & EstBB database	Stoke diagnosis (case)	stroke
Feature 1	EstBB database	sex	Sex
Feature 2	EstBB database	birth_year	Year of birth
Feature 3	EstBB database	age_at_measurement	Age during the last measurment (+/- 1 year)
Feature 4	EHR database epicrisis documents	aB.pH	pH of Arterial blood
Feature 5	EHR database epicrisis documents	aB.pO2./.FiO2	Horowitz index in Arterial blood
Feature 6	EHR database epicrisis documents	B.Baso.%	Basophils/100 leukocytes in Blood by Automated count
Feature 7	EHR database epicrisis documents	B.Baso.#	Basophils [#/volume] in Blood by Automated count
Feature 8	EHR database epicrisis documents	B.Basofiilid..#	Basophils [#/volume] in Blood
Feature 9	EHR database epicrisis documents	B.CBC.5Diff.ALP	Complete blood count (hemogram) panel in Blood by Automated count
Feature 10	EHR database epicrisis documents	B.CBC.5Diff.Eo	Complete blood count (hemogram) panel in Blood by Automated count
Feature 11	EHR database epicrisis documents	B.CBC.5Diff.Eo.%	Complete blood count (hemogram) panel in Blood by Automated count
Feature 12	EHR database epicrisis documents	B.CBC.5Diff.Prot	Complete blood count (hemogram) panel in Blood by Automated count
Feature 13	EHR database epicrisis documents	B.CBC.B.GPR	Complete blood count (hemogram) panel in Blood by Automated count
Feature 14	EHR database epicrisis documents	B.CBC.B.GRAN	Complete blood count (hemogram) panel in Blood by Automated count
Feature 15	EHR database epicrisis documents	B.CBC.B.LPR	Complete blood count (hemogram) panel in Blood by Automated count
Feature 16	EHR database epicrisis documents	B.CBC.B.LYMPH	Complete blood count (hemogram) panel in Blood by Automated count
Feature 17	EHR database epicrisis documents	B.CBC.B.MID	Complete blood count (hemogram) panel in Blood by Automated count
Feature 18	EHR database epicrisis documents	B.CBC.B.MPR	Complete blood count (hemogram) panel in Blood by Automated count
Feature 19	EHR database epicrisis documents	B.CBC.prot	Complete blood count (hemogram) panel in Blood by Automated count
Feature 20	EHR database epicrisis documents	B.Eo.%	Eosinophils/100 leukocytes in Blood by Automated count
Feature 21	EHR database epicrisis documents	B.Eo.#	Eosinophils [#/volume] in Blood by Automated count
Feature 22	EHR database epicrisis documents	B.Eosinofiilid..#	Eosinophils [#/volume] in Blood
Feature 23	EHR database epicrisis documents	B.ESR	Erythrocyte sedimentation rate by Westergren method
Feature 24	EHR database epicrisis documents	B.Hb	Hemoglobin [Mass/volume] in Blood
Feature 25	EHR database epicrisis documents	B.Hb. (set 2*)	Hemoglobin [Mass/volume] in Blood
Feature 26	EHR database epicrisis documents	B.HbA1c	Hemoglobin A1c/Hemoglobin.total in Blood
Feature 27	EHR database epicrisis documents	B.HbA1c. (set 2*)	Hemoglobin A1c/Hemoglobin.total in Blood
Feature 28	EHR database epicrisis documents	B.Hct	Hematocrit [Volume Fraction] of Blood by Automated count
Feature 29	EHR database epicrisis documents	B.Hct. (set 2*)	Hematocrit [Volume Fraction] of Blood by Automated count
Feature 30	EHR database epicrisis documents	B.IG.%	Granulocytes Immature/100 leukocytes in Blood
Feature 31	EHR database epicrisis documents	B.IG.#	Granulocytes Immature [#/volume] in Blood
Feature 32	EHR database epicrisis documents	B.IPF	Platelets reticulated/100 platelets in Blood by Automated count
Feature 33	EHR database epicrisis documents	B.IRF	Reticulocytes.immature/Reticulocytes.total in Blood
Feature 34	EHR database epicrisis documents	B.Kepptuumsed.neutrofiilid..#	Band form neutrophils [#/volume] in Blood
Feature 35	EHR database epicrisis documents	B.LCR	Platelets Large/Platelets in Blood by Automated count
Feature 36	EHR database epicrisis documents	B.Lymph.%	Lymphocytes/100 leukocytes in Blood by Automated count
Feature 37	EHR database epicrisis documents	B.Lymph.#	Lymphocytes [#/volume] in Blood by Automated count
Feature 38	EHR database epicrisis documents	B.MCH	Erythrocyte mean corpuscular hemoglobin [Entitic mass] by Automated count
Feature 39	EHR database epicrisis documents	B.MCH. (set 2*)	Erythrocyte mean corpuscular hemoglobin [Entitic mass] by Automated count
Feature 40	EHR database epicrisis documents	B.MCHC	Erythrocyte mean corpuscular hemoglobin concentration [Mass/volume] by Automated count
Feature 41	EHR database epicrisis documents	B.MCHC. (set 2*)	Erythrocyte mean corpuscular hemoglobin concentration [Mass/volume] by Automated count
Feature 42	EHR database epicrisis documents	B.MCV	Erythrocyte mean corpuscular volume [Entitic volume] by Automated count
Feature 43	EHR database epicrisis documents	B.Mono.%	Monocytes/100 leukocytes in Blood by Automated count
Feature 44	EHR database epicrisis documents	B.Mono.#	Monocytes [#/volume] in Blood by Automated count
Feature 45	EHR database epicrisis documents	B.MPV	Platelet mean volume [Entitic volume] in Blood by Automated count
Feature 46	EHR database epicrisis documents	B.Mxd.%	Basophils+Eosinophils+Monocytes/100 leukocytes in Blood by Automated count
Feature 47	EHR database epicrisis documents	B.Mxd.#	Basophils+Eosinophils+Monocytes [#/volume] in Blood by Automated count
Feature 48	EHR database epicrisis documents	B.Neut.%	Neutrophils/100 leukocytes in Blood by Automated count
Feature 49	EHR database epicrisis documents	B.Neut.#	Neutrophils [#/volume] in Blood by Automated count
Feature 50	EHR database epicrisis documents	B.Neut.#. (set 2*)	Neutrophils [#/volume] in Blood by Automated count
Feature 51	EHR database epicrisis documents	B.Pct	Plateletocrit [Volume Fraction] in Blood
Feature 52	EHR database epicrisis documents	B.PDW	Platelet distribution width [Entitic volume] in Blood by Automated count
Feature 53	EHR database epicrisis documents	B.PDW.CV	Platelet distribution width [Ratio] in Blood
Feature 54	EHR database epicrisis documents	B.Plt	Platelets [#/volume] in Blood by Automated count
Feature 55	EHR database epicrisis documents	B.RBC	Erythrocytes [#/volume] in Blood by Automated count
Feature 56	EHR database epicrisis documents	B.RDW.CV	Erythrocyte distribution width [Ratio] by Automated count
Feature 57	EHR database epicrisis documents	B.RDW.CV (set 3*)	Erythrocyte distribution width [Ratio] by Automated count
Feature 58	EHR database epicrisis documents	B.RDW.SD	Erythrocyte distribution width [Entitic volume] by Automated count
Feature 59	EHR database epicrisis documents	B.Ret.%	Reticulocytes/100 erythrocytes in Blood by Automated count
Feature 60	EHR database epicrisis documents	B.Ret.#	Reticulocytes [#/volume] in Blood by Automated count
Feature 61	EHR database epicrisis documents	B.RetHb	Hemoglobin [Entitic mass] in Reticulocytes
Feature 62	EHR database epicrisis documents	B.Segmenttuumsed.neutrofiilid..%	Neutrophils.segmented/100 leukocytes in Blood
Feature 63	EHR database epicrisis documents	B.WBC	Leukocytes [#/volume] in Blood by Automated count
Feature 64	EHR database epicrisis documents	eGFR	Glomerular filtration rate/1.73 sq M.predicted by Creatinine-based formula (MDRD)
Feature 65	EHR database epicrisis documents	eGFR..CKD.EPI.	Glomerular filtration rate/1.73 sq M.predicted by Creatinine-based formula (CKD-EPI)
Feature 66	EHR database epicrisis documents	fS.fP.Gluc	Fasting glucose [Moles/volume] in Serum or Plasma
Feature 67	EHR database epicrisis documents	fS.fP.Trigl	Fasting triglycerides [Moles/volume] in Serum or Plasma
Feature 68	EHR database epicrisis documents	INR	INR in Blood by Coagulation assay
Feature 69	EHR database epicrisis documents	P.APTT	Activated partial thrombplastin time (aPTT) in Platelet poor plasma by Coagulation assay
Feature 70	EHR database epicrisis documents	P.D.Di	Fibrin D-dimer FEU [Mass/volume] in Platelet poor plasma
Feature 71	EHR database epicrisis documents	P.PT	Prothrombin time (PT)
Feature 72	EHR database epicrisis documents	P.PT.INR	Prothrombin time (PT)
Feature 73	EHR database epicrisis documents	P.PT.%	Prothrombin activity actual/normal in Platelet poor plasma by Coagulation assay
Feature 74	EHR database epicrisis documents	S.P.ALAT	Alanine aminotransferase [Enzymatic activity/volume] in Serum or Plasma by Without P-5'-P
Feature 75	EHR database epicrisis documents	S.P.Alb	Albumin [Mass/volume] in Serum or Plasma by Bromcresol green (BCG) dye binding method
Feature 76	EHR database epicrisis documents	S.P.ALP	Alkaline phosphatase [Enzymatic activity/volume] in Serum or Plasma
Feature 77	EHR database epicrisis documents	S.P.Amyl	Amylase [Enzymatic activity/volume] in Serum or Plasma
Feature 78	EHR database epicrisis documents	S.P.ASAT	Aspartate aminotransferase [Enzymatic activity/volume] in Serum or Plasma
Feature 79	EHR database epicrisis documents	S.P.Bil	Bilirubin [Moles/volume] in Serum or Plasma
Feature 80	EHR database epicrisis documents	S.P.Bil.conj	Bilirubin.direct [Moles/volume] in Serum or Plasma

Feature 81	EHR database epicrisis documents	S.P.BNP	Natriuretic peptide B [Mass/volume] in Serum or Plasma
Feature 82	EHR database epicrisis documents	S.P.Ca	Calcium [Moles/volume] in Serum or Plasma
Feature 83	EHR database epicrisis documents	S.P.CA.125	Cancer Ag 125 [Units/volume] in Serum or Plasma
Feature 84	EHR database epicrisis documents	S.P.CA.19.9	Cancer Ag 19-9 [Units/volume] in Serum or Plasma
Feature 85	EHR database epicrisis documents	S.P.CEA	Carcinoembryonic Ag [Mass/volume] in Serum or Plasma
Feature 86	EHR database epicrisis documents	S.P.Chol	Cholesterol [Moles/volume] in Serum or Plasma
Feature 87	EHR database epicrisis documents	S.P.CK	Creatine kinase [Enzymatic activity/volume] in Serum or Plasma
Feature 88	EHR database epicrisis documents	S.P.CK.MB	Creatine kinase.MB [Enzymatic activity/volume] in Serum or Plasma
Feature 89	EHR database epicrisis documents	S.P.CK.MBm	Creatine kinase.MB [Mass/volume] in Serum or Plasma
Feature 90	EHR database epicrisis documents	S.P.Cl	Chloride [Moles/volume] in Serum or Plasma
Feature 91	EHR database epicrisis documents	S.P.Crea	Creatine [Moles/volume] in Serum or Plasma
Feature 92	EHR database epicrisis documents	S.P.CRP	C reactive protein [Mass/volume] in Serum or Plasma
Feature 93	EHR database epicrisis documents	S.P.cTnT.hs	Troponin T.cardiac [Mass/volume] in Serum or Plasma by Detection limit <= 5 ng/L
Feature 94	EHR database epicrisis documents	S.P.Digox	Digoxin [Mass/volume] in Serum or Plasma
Feature 95	EHR database epicrisis documents	S.P.Fe	Iron [Moles/volume] in Serum or Plasma
Feature 96	EHR database epicrisis documents	S.P.Fer	Ferritin [Mass/volume] in Serum or Plasma
Feature 97	EHR database epicrisis documents	S.P.Fol	Folate [Moles/volume] in Blood
Feature 98	EHR database epicrisis documents	S.P.fPSA	Prostate Specific Ag Free [Mass/volume] in Serum or Plasma
Feature 99	EHR database epicrisis documents	S.P.fT3	Triiodothyronine Free [Moles/volume] in Serum or Plasma
Feature 100	EHR database epicrisis documents	S.P.fT4	Thyroxine free [Moles/volume] in Serum or Plasma
Feature 101	EHR database epicrisis documents	S.P.GGT	Gamma glutamyl transferase [Enzymatic activity/volume] in Serum or Plasma
Feature 102	EHR database epicrisis documents	S.P.Gluc	Glucose [Moles/volume] in Serum or Plasma
Feature 103	EHR database epicrisis documents	S.P.HDL.Chol	Cholesterol in HDL [Moles/volume] in Serum or Plasma
Feature 104	EHR database epicrisis documents	S.P.iCa	Calcium.ionized [Moles/volume] in Serum or Plasma by Ion-selective membrane electrode (ISE)
Feature 105	EHR database epicrisis documents	S.P.IgG	IgG [Mass/volume] in Serum
Feature 106	EHR database epicrisis documents	S.P.IgM	IgM [Mass/volume] in Serum
Feature 107	EHR database epicrisis documents	S.P.K	Potassium [Moles/volume] in Serum or Plasma
Feature 108	EHR database epicrisis documents	S.P.LDH	Lactate dehydrogenase [Enzymatic activity/volume] in Serum or Plasma by Lactate to pyruvate reaction
Feature 109	EHR database epicrisis documents	S.P.LDL.Chol	Cholesterol in LDL [Moles/volume] in Serum or Plasma
Feature 110	EHR database epicrisis documents	S.P.Lip	Lipase [Enzymatic activity/volume] in Serum or Plasma
Feature 111	EHR database epicrisis documents	S.P.Mg	Magnesium [Moles/volume] in Serum or Plasma
Feature 112	EHR database epicrisis documents	S.P.Myogl	Myoglobin [Mass/volume] in Serum or Plasma
Feature 113	EHR database epicrisis documents	S.P.Na	Sodium [Moles/volume] in Serum or Plasma
Feature 114	EHR database epicrisis documents	S.P.NT.proBNP	Natriuretic peptide.B prohormone [Mass/volume] in Serum or Plasma
Feature 115	EHR database epicrisis documents	S.P.P	Phosphate [Moles/volume] in Serum or Plasma
Feature 116	EHR database epicrisis documents	S.P.PCT	Procalcitonin [Mass/volume] in Serum or Plasma
Feature 117	EHR database epicrisis documents	S.P.Prot	Protein [Mass/volume] in Serum or Plasma
Feature 118	EHR database epicrisis documents	S.P.PSA	Prostate specific Ag [Mass/volume] in Serum or Plasma
Feature 119	EHR database epicrisis documents	S.P.PTH	Parathyrin.intact [Moles/volume] in Serum or Plasma
Feature 120	EHR database epicrisis documents	S.P.Transf	Transferrin [Mass/volume] in Serum or Plasma
Feature 121	EHR database epicrisis documents	S.P.TSH	Thyrotropin [Units/volume] in Serum or Plasma
Feature 122	EHR database epicrisis documents	S.P.UA	Urate [Moles/volume] in Serum or Plasma
Feature 123	EHR database epicrisis documents	S.P.Urea	Urea [Moles/volume] in Serum or Plasma
Feature 124	EHR database epicrisis documents	S.P.Vit.B12	Cobalamin (Vitamin B12) [Mass/volume] in Serum or Plasma
Feature 125	EHR database epicrisis documents	U.Alb./.U.Crea	Microalbumin/Creatinine [Ratio] in Urine
Feature 126	EHR database epicrisis documents	U.Bil.strip	Bilirubin.total [Moles/volume] in Urine by Test strip
Feature 127	EHR database epicrisis documents	U.Crea	Creatinine [Moles/volume] in Urine
Feature 128	EHR database epicrisis documents	U.Erutrotsuudid	Erythrocytes [#/area] in Urine sediment by Microscopy high power field
Feature 129	EHR database epicrisis documents	U.Gluc.strip	Glucose [Moles/volume] in Urine by Automated test strip
Feature 130	EHR database epicrisis documents	U.Ket.strip	Ketones [Moles/volume] in Urine by Automated test strip
Feature 131	EHR database epicrisis documents	U.Lameepiteeli.cells	Epithelial cells.squamous [#/area] in Urine sediment by Microscopy high power field
Feature 132	EHR database epicrisis documents	U.Leukotsuudid	Leukocytes [#/area] in Urine sediment by Microscopy high power field
Feature 133	EHR database epicrisis documents	U.Nit.strip	Nitrite [Presence] in Urine by Automated test strip
Feature 134	EHR database epicrisis documents	U.P	Erythrocytes [#/volume] in Urine by Automated test strip
Feature 135	EHR database epicrisis documents	U.pH.strip	pH of Urine by Automated test strip
Feature 136	EHR database epicrisis documents	U.Prot	Protein [Mass/volume] in Urine
Feature 137	EHR database epicrisis documents	U.Prot.strip	Protein [Mass/volume] in Urine by Automated test strip
Feature 138	EHR database epicrisis documents	U.Prot.strip. (set 2*)	Protein [Mass/volume] in Urine by Automated test strip
Feature 139	EHR database epicrisis documents	U.RBC.strip	Erythrocytes [#/volume] in Urine by Automated test strip
Feature 140	EHR database epicrisis documents	U.Sed.m.panel	Urinalysis complete panel in Urine
Feature 141	EHR database epicrisis documents	U.SG.strip	Specific gravity of Urine by Automated test strip
Feature 142	EHR database epicrisis documents	U.Strip	Urinalysis dipstick panel in Urine by Automated test strip
Feature 143	EHR database epicrisis documents	U.Transitoorse.epiteeli.cells	Transitional cells [#/area] in Urine sediment by Microscopy high power field
Feature 144	EHR database epicrisis documents	U.Ubg.strip	Urobilinogen [Moles/volume] in Urine by Automated test strip
Feature 145	EHR database epicrisis documents	U.WBC.strip	Leukocytes [#/volume] in Urine by Automated test strip
Feature 146	EHR database epicrisis documents	U.WBC.strip.ord	Leukocytes [Presence] in Urine
Feature 147	EHR database epicrisis documents	U.WBC.strip.ord. (set 2*)	Leukocytes [Presence] in Urine
Feature 148	EHR database epicrisis documents	XXX.Monotsuudid	Monocytes
Feature 149	Calculated from data	count_na	Number of LOINC measurments missing
Feature 150	Calculated from data	count_analytes	Number of LOINC measurments that have value
Feature 151	EstBB database	ICD-10	Last ICD-10 diagnosis code available (except for I60, I61, I62, I63, I64)

* The sets are clinical parameters measured in different units (or methodology). Due to the database connecting all Estonian laboratory systems the units and methods may vary between different locations and times of measurement.

The Target variable IS is predicted using the 151 features. For every feature the data source is given (either EHR epicrisis records or EstBB database). Feature names are explained by the description column.

Fig. S21. Workflow of ML model creation and testing.

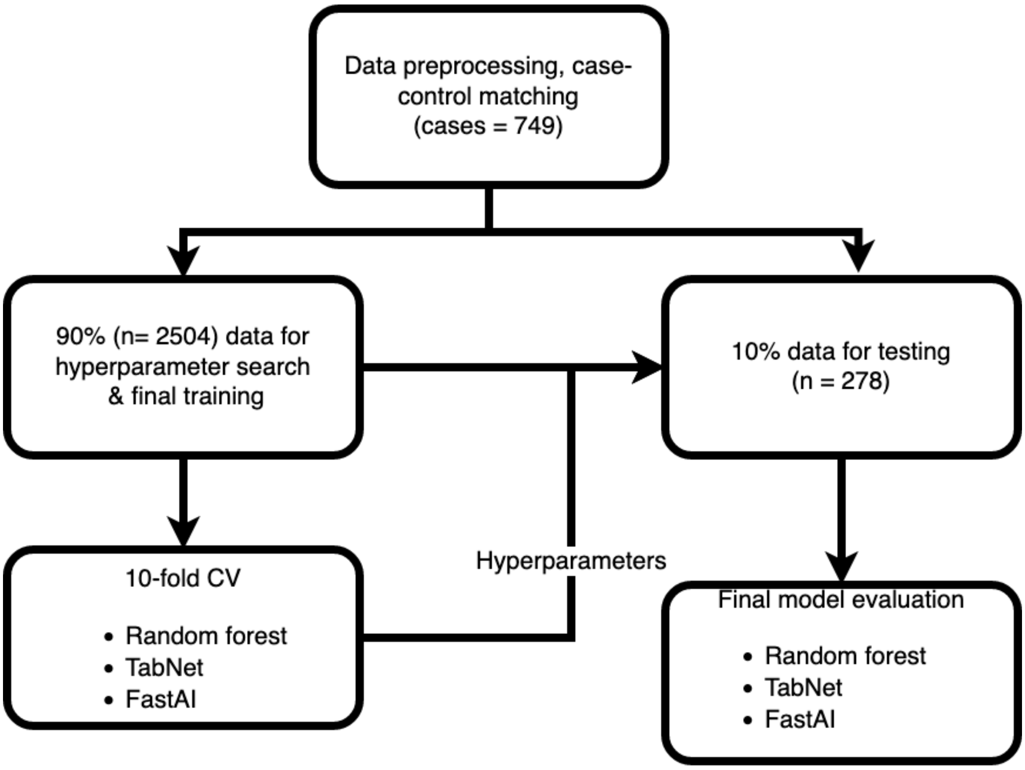

The test data set is used only for the final evaluation of the models and is not used in the training or hyper-parameter selection process. As selecting the right hyper-parameters for the data is crucial for good performance of the model this is solved with using 10-fold cross validation.

Table S22. The ML ensemble models tested.

Model	AUC
RF (original)	0.942
FastAI (original)	0.879
TabNet (original)	0.904
(RF+ TabNet + FastAI) / 3	0.943
(3*RF + 2* TabNet + FastAI) / 6	0.945
(3*RF + TabNet + FastAI) / 5	0.947
(2* RF + FastAI) / 3	0.947
(3* RF + FastAI) / 4	0.948
(4* RF + FastAI) / 5	0.949
(5* RF + FastAI) / 6	0.948

Enseble models using all 3 trained ML models were created such that the average weighted prediction score was calculated using different set of weights. As DNN predictions tend to be overconfident the large weight score for RF model helps to balance the ensemble prediction. AUC values of the different ensemble models tested are shown in the table.
